# Potent cross-reactive antibodies following Omicron breakthrough in vaccinees

**DOI:** 10.1016/j.cell.2022.05.014

**Published:** 2022-06-09

**Authors:** Rungtiwa Nutalai, Daming Zhou, Aekkachai Tuekprakhon, Helen M. Ginn, Piyada Supasa, Chang Liu, Jiandong Huo, Alexander J. Mentzer, Helen M.E. Duyvesteyn, Aiste Dijokaite-Guraliuc, Donal Skelly, Thomas G. Ritter, Ali Amini, Sagida Bibi, Sandra Adele, Sile Ann Johnson, Bede Constantinides, Hermione Webster, Nigel Temperton, Paul Klenerman, Eleanor Barnes, Susanna J. Dunachie, Derrick Crook, Andrew J. Pollard, Teresa Lambe, Philip Goulder, OPTIC consortium, ISARIC4C consortium, Neil G. Paterson, Mark A. Williams, David R. Hall, Juthathip Mongkolsapaya, Elizabeth E. Fry, Wanwisa Dejnirattisai, Jingshan Ren, David I. Stuart, Gavin R. Screaton

**Affiliations:** 1 Wellcome Centre for Human Genetics, Nuffield Department of Medicine, University of Oxford, Oxford, UK; 2 Division of Structural Biology, Nuffield Department of Medicine, University of Oxford, The Wellcome Centre for Human Genetics, Oxford, UK; 3 Chinese Academy of Medical Science (CAMS) Oxford Institute (COI), University of Oxford, Oxford, UK; 4 Diamond Light Source Ltd, Harwell Science & Innovation Campus, Didcot, UK; 5 Oxford University Hospitals NHS Foundation Trust, Oxford, UK; 6 Peter Medawar Building for Pathogen Research, Oxford, UK; 7 Nuffield Department of Clinical Neurosciences, University of Oxford, Oxford, UK; 8 Translational Gastroenterology Unit, University of Oxford, Oxford, UK; 9 Oxford Vaccine Group, Department of Paediatrics, University of Oxford, Oxford, UK; 10 Nuffield Department of Medicine, University of Oxford, Oxford, UK; 11 Viral Pseudotype Unit, Medway School of Pharmacy, University of Kent and Greenwich, Chatham Maritime, Kent ME4 4TB, UK; 12 NIHR Oxford Biomedical Research Centre, Oxford, UK; 13 Centre For Tropical Medicine and Global Health, Nuffield Department of Medicine, University of Oxford, Oxford, UK; 14 Mahidol-Oxford Tropical Medicine Research Unit, Bangkok, Thailand; 15 Department of Paediatrics, University of Oxford, Oxford, UK

**Keywords:** SARS-CoV-2, COVID-19, Omicron, BA.1, BA.1.1, BA.2, antibody responses, crystallography, variants of concern, receptor binding domain, neutralization, immune escape

## Abstract

Highly transmissible Omicron variants of SARS-CoV-2 currently dominate globally. Here, we compare neutralization of Omicron BA.1, BA.1.1, and BA.2. BA.2 RBD has slightly higher ACE2 affinity than BA.1 and slightly reduced neutralization by vaccine serum, possibly associated with its increased transmissibility. Neutralization differences between sub-lineages for mAbs (including therapeutics) mostly arise from variation in residues bordering the ACE2 binding site; however, more distant mutations S371F (BA.2) and R346K (BA.1.1) markedly reduce neutralization by therapeutic antibody Vir-S309. In-depth structure-and-function analyses of 27 potent RBD-binding mAbs isolated from vaccinated volunteers following breakthrough Omicron-BA.1 infection reveals that they are focused in two main clusters within the RBD, with potent right-shoulder antibodies showing increased prevalence. Selection and somatic maturation have optimized antibody potency in less-mutated epitopes and recovered potency in highly mutated epitopes. All 27 mAbs potently neutralize early pandemic strains, and many show broad reactivity with variants of concern.

## Highlights

• Potent RBD antibodies from Omicron breakthrough vaccinees broadly neutralize VoC
• These, possible recall antibodies, are focused in two main clusters
• Somatic maturation adapts public antibodies to recover potency
• BA.2 > BA.1 ACE2 affinity. BA.2 < BA.1 neutralization by vaccine serum and Vir-S309

## Graphical abstract

**Figure:**
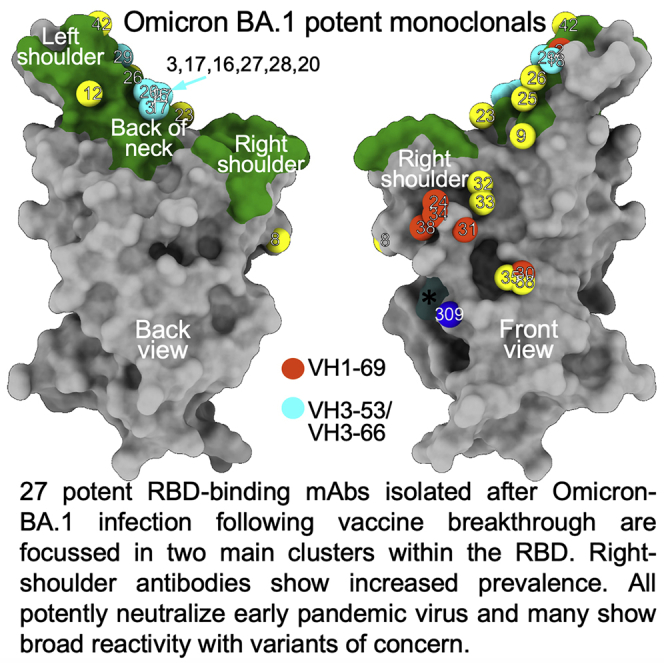

## Teaser

Analysis of antibodies from SARS-CoV-2 Omicron breakthrough infections reveals their structural and functional properties as well as ability to neutralize different pandemic strains.

## Introduction

Omicron BA.1 was first reported in late November 2021 in Southern Africa and spread explosively around the world, becoming the dominant SARS-CoV-2 variant in the UK by December 17th (https://assets.publishing.service.gov.uk/government/uploads/system/uploads/attachment_data/file/1042100/20211217_OS_Daily_Omicron_Overview.pdf). Omicron (where not specified, Omicron refers to sub-lineage BA.1) contains an unprecedented number of mutations concentrated in the Spike (S) gene which carries 30 substitutions plus the deletion of 6 and insertion of 3 residues.

S is the major surface glycoprotein on the SARS-CoV-2 virion and is involved in viral attachment to target cells via the interaction of cell-surface-expressed angiotensin-converting enzyme 2 (ACE2) with the receptor binding site, at the tip of the receptor binding domain (RBD), in the S1 fragment of S (Lan et al., 2020). Following attachment, cleavage of S releases S1, allowing a major conformational change in S2, exposing a hydrophobic loop that executes fusion of viral and host cell membranes, releasing the viral genome to initiate viral replication (Walls et al., 2017).

Since late 2020, a succession of variants of concern (VoC) have emerged. Some have caused large regional outbreaks (Beta [Zhou et al., 2021], Gamma [Dejnirattisai et al., 2021b]) whilst others have become dominant globally (Alpha [Supasa et al., 2021] then Delta [Liu et al., 2021a] then Omicron [Dejnirattisai et al., 2022]). All VoC contain mutations in the RBD, which potentially serve two functions. Firstly, to increase affinity to ACE2 and potentially increase transmissibility; this is observed for Alpha, Beta and Gamma (Dejnirattisai et al., 2021b; Supasa et al., 2021; Zhou et al., 2021). Secondly, mutations have the potential to cause escape from serum induced by vaccines or previous SARS-CoV-2 infection. Escape from neutralization is modest for Alpha; more marked for Beta, Gamma, and Delta; and more extreme for Omicron (Dejnirattisai et al., 2022; Dejnirattisai et al., 2021a; Dejnirattisai et al., 2021b; Liu et al., 2021a; Supasa et al., 2021; Zhou et al., 2021).

The extensive mutational burden in Omicron S disrupts the activity of the majority of potent neutralizing mAbs, leading to severe knockdown or complete loss of the neutralizing capacity of serum from natural infection or vaccination, contributing to increased transmissibility and explosive spread (Cele et al., 2021; Dejnirattisai et al., 2022). However, it is clear that respectable anti-Omicron titres are achieved following third-dose vaccination, providing good protection from hospitalization and severe disease (Dejnirattisai et al., 2022; Mahase, 2021b).

**Figure 1:**
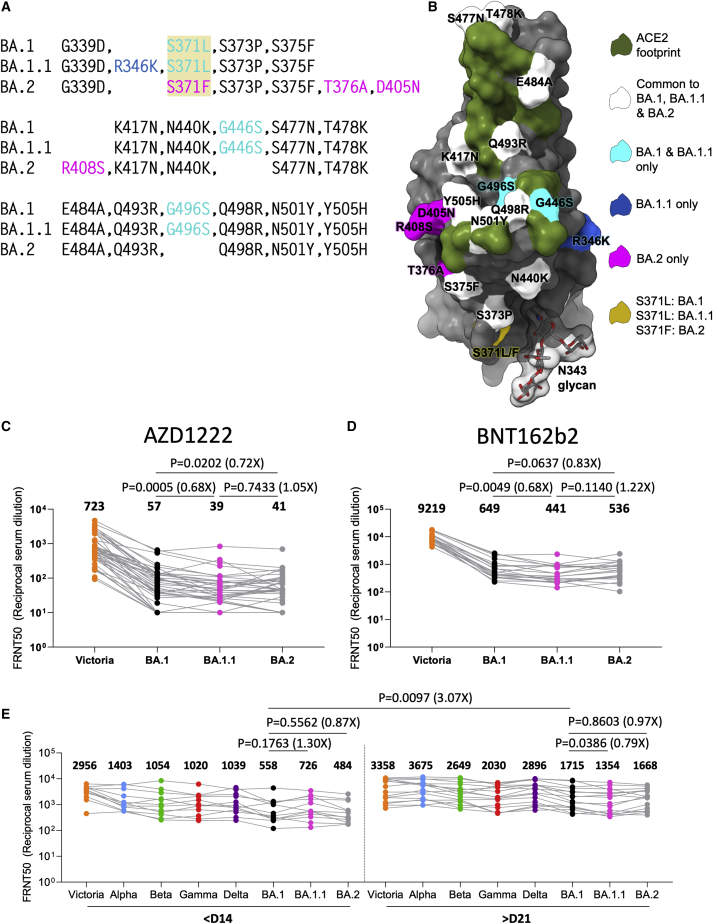
The sub-lineages of Omicron and neutralization of BA.1 and BA.2 by vaccine and Omicron serum (A) Comparison of the mutations of Omicron BA.1, BA.1.1, and BA.2 RBDs. (B) Position of these on the RBD (gray surface with the ACE2 footprint in dark green). Mutations common to all three are shown in white, those common to BA.1 and BA.1.1 in cyan, those unique to BA.1.1 in blue, and those unique to BA.2 in magenta. Residue 371 (yellow) is mutated in all Omicron viruses but differs between BA.1 and BA.2. The N343 glycan is shown in a transparent surface. (C and D) Live virus neutralization. of Victoria, BA.1, BA.1.1, and BA.2 28 days following the third doses of AZD1222 (n = 41) (C), BNT162b2 (n = 20) (D). (E) Live virus neutralization assays with VoC using sera obtained <14 days (median 13 days) and >21 days (median 38 days) following symptom onset. Geometric mean titers are shown above each column. The Wilcoxon matched-pairs signed rank test (C and D) and Mann-Whitney test (E) were used and two-tailed P values calculated.

As of February 2022, two sub-lineages additional to BA.1 have been identified: BA.1.1 and BA.2 (https://www.who.int/publications/m/item/weekly-epidemiological-update-on-covid-19---1-february-2022). Compared to BA.1, BA.1.1 contains an additional R346K mutation (it is thus also known as BA.1+R346K), whilst BA.2 bears 8 unique mutations in S (6 within the RBD, Figure 1A) and lacks 13 mutations found in BA.1. BA.2 is now becoming dominant in several countries (https://www.nature.com/articles/d41586-022-00471-2) and is estimated to account for approximately 93.7% of cases in England (https://www.gov.uk/government/news/covid-19-variants-identified-in-the-uk).

Here we investigate the Omicron sub-lineages BA.1.1 and BA.2 in addition to BA.1. We report slightly increased affinity of BA.2 RBD for ACE2. We show that BA.1.1 and BA.2 are modestly more difficult to neutralize than BA.1 using vaccine serum. Concerningly, a number of mAbs, including those in clinical use (Chen et al., 2021; Mahase, 2021a; Weinreich et al., 2021), show marked differential sensitivity to BA.1 or BA.2 for which we provide structural explanations. We describe the generation of a panel of 545 mAbs from volunteers following vaccine break-through Omicron infections and perform detailed analysis of the 28 most potent (IC50 < 100 ng/mL), which all potently neutralized early pandemic SARS-CoV-2 strain Victoria and were more heavily mutated than mAbs obtained from primary infections, consistent with them having been recalled and adapted from the response to vaccination. Many are fully cross-reactive amongst early pandemic and all VoC (Victoria, Alpha, Beta, Gamma, Delta, and Omicron).

## Results

### Omicron BA.2 lineage

BA.2 shares 21 amino acid substitutions with BA.1, spread throughout S (Figure 1A); however, BA.1 has an additional 6 amino acid deletions, 3 insertions, and 9 substitutions compared to BA.2, whilst BA.2 has an additional 3 deletions and 7 substitutions compared to BA.1. In the RBD, BA.1 contains unique mutations S371L, G446S, and G496S and in some isolates R346K (BA.1.1), while BA.2 carries S371F, T376A, D405N, and R408S (Figures 1A and 1B). All of these mutations have the potential to differentially affect antibody binding and could modulate neutralization, particularly BA.1 G446S, G496S and BA.2 D405N, R408S, which lie at the edge of the ACE2 binding footprint. Residue 371 (which differs between BA.1 [Leu] and BA.2 [Phe]) and the BA.1.1 specific R346K change lie close to the N343 glycan and could modulate binding of potent antibodies to this region (Figure 1B). Interestingly, the sub-lineage specific mutations segregate, with BA.1 and BA.1.1 changes lying on one side of the ACE2 footprint and BA.2 changes on the other side (Figure 1B), possibly reflecting different selective pressure on the BA.1 and BA.2 sub-lineages.

### Neutralization of BA.1, BA.1.1, and BA.2 by immune sera

To assess differential sensitivity to neutralization of the Omicron sub-lineages, we performed neutralization assays on Victoria (an early pandemic isolate containing an S247R substitution in the S NTD compared to the Wuhan vaccine strain), together with BA.1, BA.1.1, and BA.2 viruses using sera collected from vaccinees 28 days following third doses of the Oxford/AstraZeneca AZD1222 (n = 41) or Pfizer/BioNtech BNT162b2 (n = 20) vaccines (Figures 1C and 1D).

There was a major reduction in neutralization titre for all Omicron viruses for both vaccines. For AZD1222 vaccinees, BA.1.1 and BA.2 showed small but significant reductions in titers relative to BA.1; BA.1 vs. BA.1.1, 1.5-fold reduction (p = 0.0005) and BA.1 vs. BA.2 1.4-fold reduction (p = 0.0202). BNT162b2, following the third vaccine dose, showed the same trend; BA.1 vs. BA.1.1, 1.5-fold reduction (p = 0.0049) and BA.1 vs. BA.2, 1.2-fold reduction (p = 0.0637) (Figures 1C and 1D).

**Figure S1:**
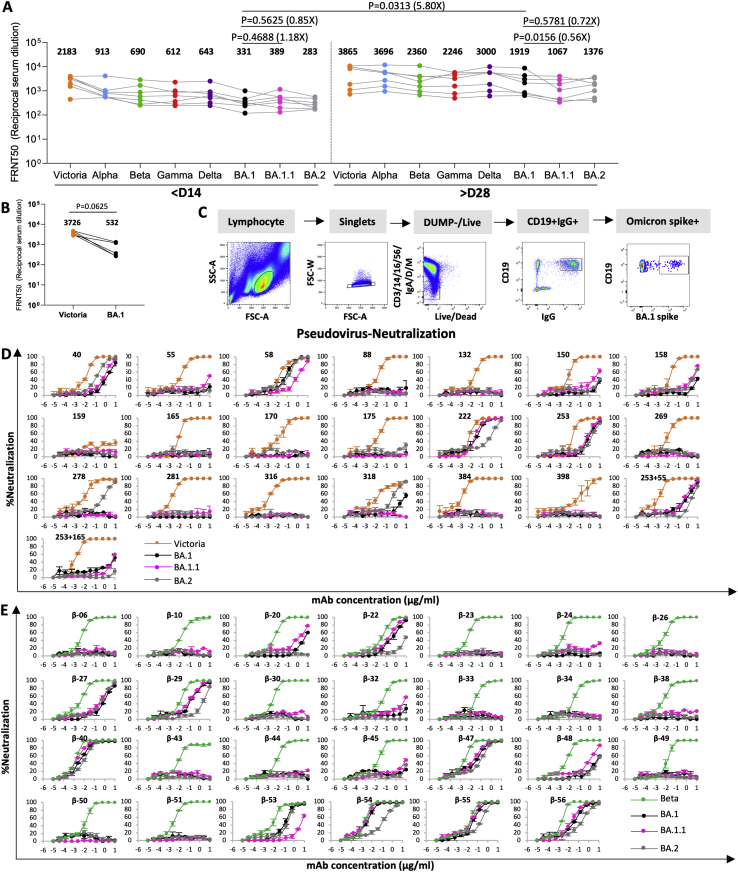
mAb production and neutralization curves for BA.1, BA.1.1 and BA.2 (A) Live virus neutralization of paired samples taken early and late following Omicron infection. Geometric mean titers are shown above each column. The Wilcoxon matched-pairs signed rank test was used for the analysis and two-tailed P values were calculated. (B) FRNT50 titers against Victoria and Omicron BA.1 from donors for the production of Omicron mAb are shown. (C) FACS plots showing the sorting of B cells using full length Omicron S. (D and E) (D) early pandemic mAb and (E) Beta mAb. Related to Figure 3.

Next, we looked at the neutralization profile across all VoC for serum collected from cases infected with BA.1. Early samples (n = 12) were taken ≤14 days from symptom onset (median 13 days); later samples (n = 16) were taken ≥21 days following symptom onset (median 38 days). All cases had received at least 2 doses of vaccine (4 AZD1222, 16 BNT162b2, and 1 Johnson & Johnson JNJ-78436735), and 3 of the late convalescent cases received a third dose of vaccine following Omicron infection. Neutralization was tested using live virus assays (Figure 1E). At early time points, as expected, all vaccinated cases had high titers to Victoria with geometric mean FRNT50 close to 1/3,000 and exhibited broad neutralization of VoC with FRNT50 > 1/1,000 for all viruses except Omicron (FRNT50 = 558). At the later time point, titers were increased against all variants including BA.1 (3.1-fold p = 0.0097), although titers to Victoria were only modestly increased. Comparison of early and late samples taken from the same individuals confirmed the broad boosting of the response following Omicron infection (Figure S1A).

### Potently neutralizing antibodies isolated following Omicron infection

We generated a panel of human monoclonal antibodies from volunteers who had recovered from sequence confirmed BA.1 infection having previously received 2 doses of the Pfizer-BioNtech vaccine. First, we performed neutralization assays against BA.1 and Victoria. In all cases, the BA.1 neutralization titre, measured by the serum dilution required to reduce virus foci by 50% (FRNT50), was above 100 (Figure S1B).

B cells from 5 donors were stained with full-length BA.1 S trimer and single cells sorted by FACS (Figure S1C). Following a degenerate RT-PCR reaction, heavy and light chain sequences were assembled into expression vectors using the Gibson reaction and transfected into 293T cells. Culture supernatants were screened for reactivity to full length BA.1 or WT S (wild-type Wuhan) together with BA.1 RBD and NTD. In total, 1,122 single cells were sorted and 545 mAbs recovered.

**Figure 2:**
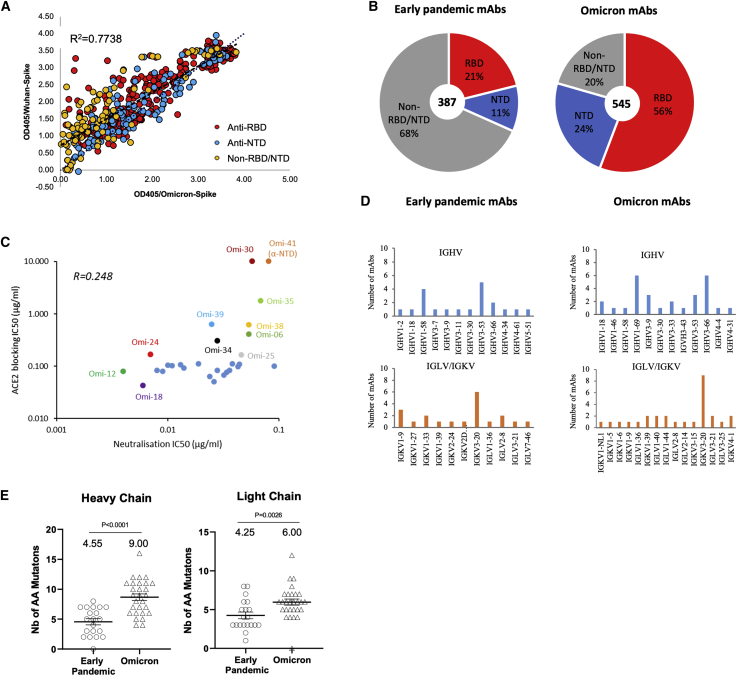
Generation of a panel of Omicron mAbs (A) ELISA of 525 mAb comparing OD against Wuhan and BA.1 S trimer, further mapping to RBD (red), NTD (blue) and non-RBD/NTD (orange) is indicated. (B) Proportion of RBD and NTD binding antibodies found in the Omicron mAb compared to early pandemic mAb. (C) Effect of mAb on binding of ACE2 to BA.1 S trimer. (D) Heavy- and light-chain variable gene usage. (E) Somatic mutations found in the potent Omicron mAb (FRNT50 < 100 ng/mL) compared to the early pandemic set. See also Table S1.

Almost all mAbs cross-reacted between WT and BA.1 S by ELISA (Figure 2A). Compared with a previous panel of monoclonal antibodies we produced from naive cases infected early during the pandemic, we found a higher proportion of RBD-reactive mAbs: 56% compared to 21% (binomial two-population proportion test, p < 0.0001, Z∼10) (Figure 2B). Underscoring this, in a similar study on early pandemic samples (Zost et al., 2020), raw data on unsorted B-cells showed a similar proportion (23%) of RBD-reactive mAbs. Some 50% of the remaining antibodies (129/545) bound the NTD.

### Characterization of the most potent Omicron monoclonal antibodies

Neutralization assays were used to select the 28 most potent antibodies, with BA.1 FRNT50 titers <100 ng/mL. All but one of these bound the RBD (Omi-41 bound the NTD), but none cross-reacted with SARS-CoV-1 S protein by ELISA. With the exception of Omi-30 and Omi-41, they reduce the interaction of RBD with ACE2 (Figure 2C). However, several IGHV1-69 antibodies were less effective blockers (Figure 2C).

Examination of the heavy chain gene family usage (Figure 2D; Table S1) revealed that Omi-32 and Omi-33, which differed by 5 amino acids, were clonally related (IGHV3-33). 30% (9/28) of the monoclonals belong to the IGHV3-53 and related IGHV3-66 gene families. These antibodies generally bind a site at the back of the neck of the RBD and block ACE2 binding (Dejnirattisai et al., 2021a). They form the best-known public antibody response to SARS-CoV-2 infection (Yuan et al., 2020; Dejnirattisai et al., 2021a, Liu et al., 2021b) with a similar incidence (7/20) seen in potent early pandemic antibodies (Dejnirattisai et al., 2021a). However, those raised against early pandemic virus have little activity on VoC containing the N501Y mutation (Alpha, Beta, Gamma [Supasa et al., 2021]). We previously described IGHV3-53 antibodies (mAb 222 and Beta-27) resistant to the N501Y change (Dejnirattisai et al., 2021b), but even these show little activity to BA.1 or BA.2 (Figures S1D and S1E) (Dejnirattisai et al., 2021b, 2022).

Roughly one-half of the gene families we observed in the potent early pandemic antibodies are also represented in the Omicron set (Figure 2D). Although IGHV1-69 did not feature in our potent early antibodies, it has been seen by others in a number of potent mAbs isolated following natural infection or vaccination (Wang et al., 2021; Andreano et al., 2021; Cho et al., 2021). We found 6 IGHV1-69 antibodies (2, 24, 30, 31, 34, and 38) out of 27 potent RBD binders.

We found higher levels of somatic mutation in both heavy and light chains of Omicron mAbs than in the early pandemic set of antibodies; mean number of amino acid substitutions 9.00/6.00 for Omicron and 4.55/4.25 for early pandemic (p < 0.0001 and p = 0.0026) for heavy and light chains, respectively (Figure 2E).

**Figure S2:**
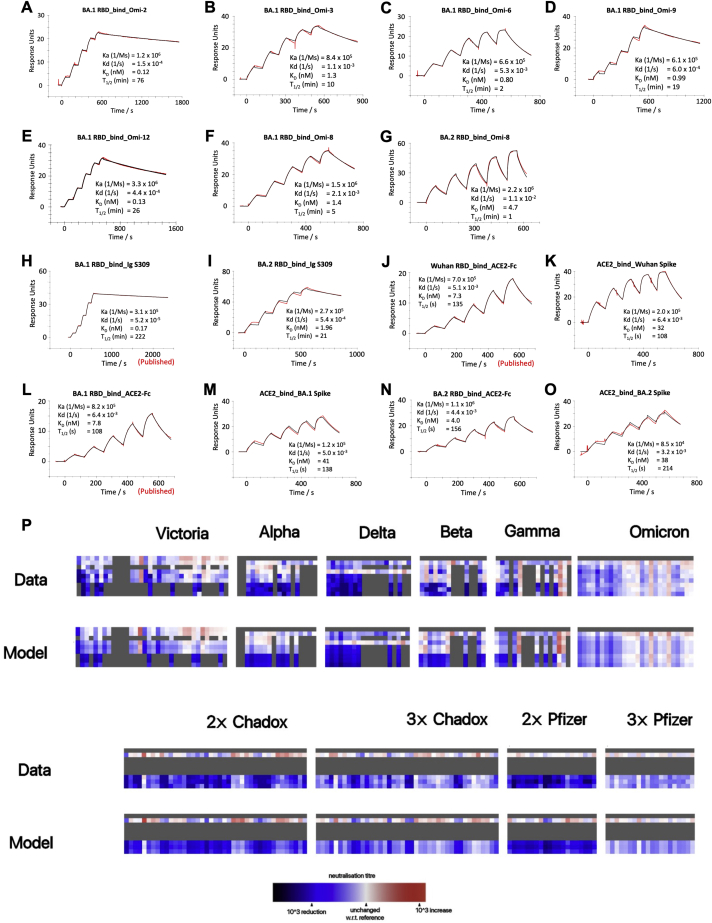
Surface plasmon resonance measurements, Antigenic map calculation (A–O) SPR traces for the indicated BA.1 or BA.2 binding to the indicated mAb or ACE2. (P) Neutralization data and model (log titre values) used to calculate antigenic maps in Figures 5 and 7E. Columns represent sera collected from inoculated volunteers or infected patients. Rows are challenge strains: Victoria, Alpha, Delta, Beta, Gamma, BA.1, BA1.1 and BA.2 in order. Values are colored according to their deviation from the reference value; the reference value is calculated on a serum-type basis as the average of neutralization titers from the row which gives this the highest value, Related to Figure 5.

The potency of these antibodies is underscored by SPR measurements of the binding of 6 selected mAbs to BA.1 RBD. The antibodies bind very tightly with affinities between 5 nM and 120 pM (Figures S2A–S2F; for clarity, SPR results are grouped in Figures S2A–S2O).

### Broad neutralization of VoC by potent Omicron antibodies

**Figure 3:**
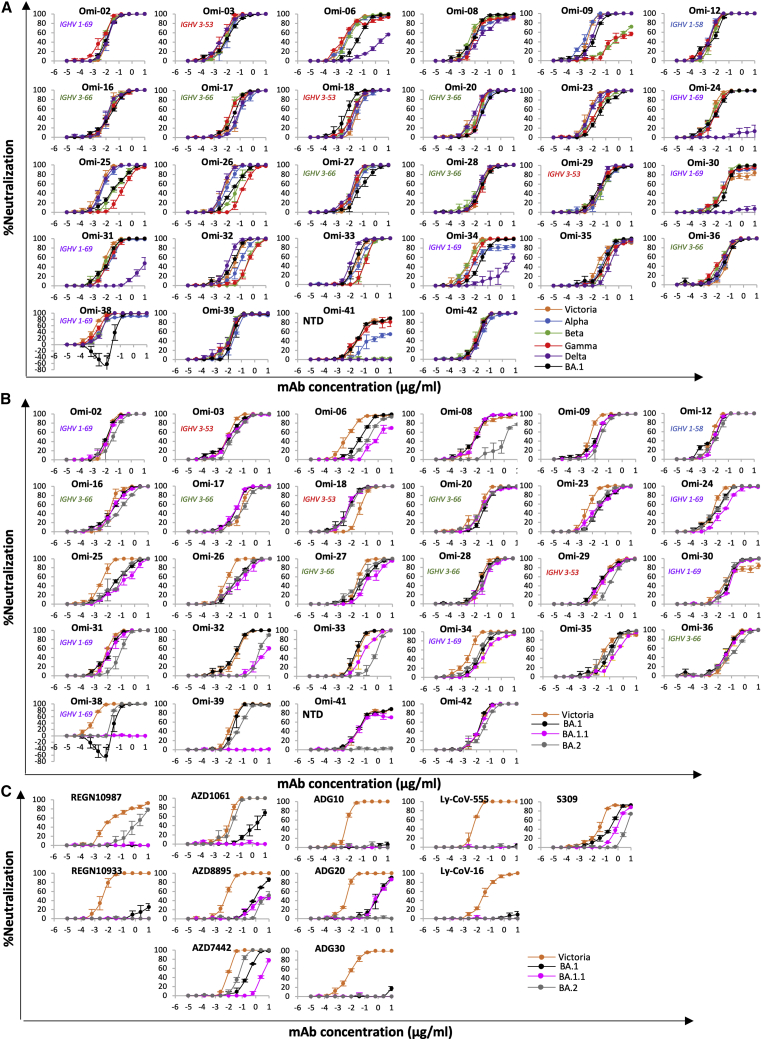
Neutralization assays against Omicron and VoC (A and B) Live virus neutralization curves using Omicron mAb (A) Victoria, Alpha, Beta, Gamma, Delta, and Omicron BA.1 viruses, (B) neutralization of Victoria, BA.1, BA.1.1, and BA.2 viruses. (C) Neutralization of Victoria, BA.1, BA.1.1, and BA.2 by antibodies being developed for commercial use. See also Figure S1 and Tables S2A and S2B.

Live virus neutralization assays show that FRNT50 titers to Victoria are <100 ng/mL for all 28 potent mAbs (Figure 3A, Table S2A), perhaps because the antibodies have been derived from vaccine-induced memory B cells. 5/28 antibodies (Omi-3, 8, 12, 18, and 24) neutralize BA.1 with FRNT50 titers <10 ng/mL (9, 8, 4, 6, and 7 ng/mL, respectively) with FRNT90 titers of 189, 101, 44, 33, and 83 ng/mL, respectively.

Live virus neutralization assays against Alpha, Beta, Gamma, and Delta VoC show 17/28 antibodies are cross-reactive against all VoC with <10-fold difference in FRNT50 titers between all viruses (Figure 3A, Table S2A). Omi-6, 24, 30, 31, 34, and 41 show reduced or absent activity against Delta, and 4 of these belong to the IGHV1-69 family, whose epitope may impinge on the L452R Delta mutation (Delta has 2 RBD mutations and shares T478K with BA.1). Antibodies Omi-9 and 32 perform poorly on Beta and Gamma and may be sensitive to E484K found in these VoC but tolerate the E484A change in Omicron (Omicron shares N501Y and K417N mutations with Beta whilst Gamma has N501Y, K417T). Interestingly, one IGHV1-69 antibody, Omi-38, showed some enhancement of BA.1 infection at lower concentrations, up to 63% higher infection than the control without antibody. This was not seen for other SARS-CoV-2 variants against Omi-38.

Finally, of 129 anti-NTD mAbs isolated, only one, Omi-41, showed FRNT50 titers <100 ng/mL. Omi-41 showed neutralizing activity against Victoria, Alpha, and Gamma but no activity against Beta and Delta, presumably reflecting the unique spectrum of NTD changes found in these viruses.

### Neutralization of Omicron sub-lineages by potent antibodies

For all 28 potent Omicron antibodies, neutralization assays of BA.1, BA.1.1, and BA.2 were performed using live virus (Figure 3B; Table S2A). Most showed little difference between BA.1, BA.1.1, and BA.2. However, there were notable exceptions; BA.2 neutralization was reduced 189-, 79-, and 26-fold compared to BA.1 for Omi-8, 32, and 33 respectively, while BA.1.1 neutralization was reduced 28- and 193-fold compared to BA.1 for Omi-6 and 32, respectively, and knocked out for Omi-38 and 39. In line with this, SPR analysis showed that binding of Omi-8 to BA.2 is 5-fold weaker than to BA.1 (Figures S2F and S2G).

Pseudoviral neutralization curves for panels of antibodies isolated from early pandemic and Beta cases against BA.1, BA.1.1, and BA.2 are shown in Figures S1D and S1E and Table S2B; in most cases, titers are similar, but mAbs 40, 278, and 318 neutralize BA.2 > BA.1, whereas early pandemic mAb 222, Beta-22, 29, 54, 55, and 56 neutralize BA.1 > BA.2, whilst Beta-53, which binds close to the N343 glycan, shows reduced neutralization of BA.1.1.

### Neutralization of Omicron sub-lineages by antibodies developed for clinical use

Neutralization assays against Victoria, BA.1, BA.1.1, and BA.2 for clinical mAbs revealed a number of differences (Figure 3C, Table S2A).

*REGN 10987 and 10933*: REGN 10933 (Weinreich et al., 2021) binds the back of the left shoulder, and 10987 binds the right shoulder. REGN10933 H2 contacts residues 484 and 493 and is sensitive to the E484K mutation. Since E484A and Q493R are present in all Omicron strains, neutralizing activity to Omicron is universally lost. REGN10987 H2 contacts residue 446 and has no activity against Omicron variants containing G446S but retains some neutralization capability against BA.2, which lacks the G446S mutation.

*AZD8895 and AZD1061*: AZD8895 and AZD1061 bind the back of the left shoulder and the front of the right shoulder, respectively. AZD1061 can neutralize BA.2 (<10-fold reduction compared to Victoria), but activity against BA.1 is markedly reduced, and neutralization of BA.1.1 is knocked out. This is due to the LC CDR2 contacting G446S in BA.1 and the R346K (BA.1.1) mutation making strong interactions with the HC CDR3. AZD8895 shows reduced neutralization due to the H2 contacts with the Q493R mutation universally present in the Omicron lineage (Figure 3C).

*LY-CoV016 and 555*: Activity of both antibodies on the entire Omicron lineage is knocked out. LY-CoV016 (IGHV3-53) makes extensive interactions with N501 and Y505 via L1 and L3, making it sensitive to mutations at these residues. LY-CoV555 (Sun and Ho, 2020) is vulnerable to the E484K mutation in Beta (Liu et al., 2021a) but likely tolerates E484A; however, contacts with the universal Omicron Q493R mutation will abrogate binding across the board.

**Figure 4:**
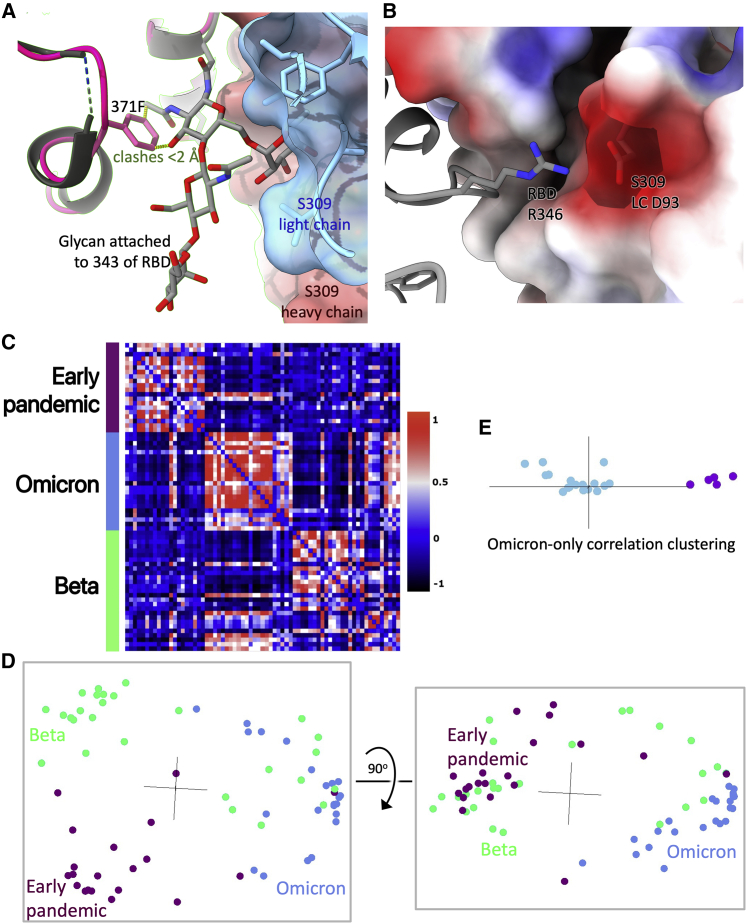
Reasons for attenuation of S309 in different Omicron sub-lineages and correlation of neutralization between antibodies from different responses (A) S309 is shown as a semi-transparent surface (heavy chain red, light chain blue) with the glycan attached to residue 343 of the RBD drawn as sticks. BA.2 RBD is shown in dark pink (Table S3A) and BA.1 RBD (PDB:7TLY) in gray. The RBD’s have been superimposed. Contacts <2.0 Å between Phe 371 and the glycan are shown as dotted lines. (B) The contact between Arg 346 of the RBD and S309 light-chain Asp 93 (PDB:7BEP). The electrostatic surface of S309 is shown. (C) Cross-correlation matrix between pairs of antibodies. Each pairwise value is the correlation coefficient between the normalized log neutralization titers of the corresponding antibodies against a panel of SARS-CoV-2 (Victoria, Alpha, Beta, Gamma, Delta, BA.1). (D) Cluster4X principal component analysis of the cross-correlation matrix in C from two orthogonal views. (E) Principal component analysis on the sub-matrix of C consisting of only the BA.1 antibodies. Omi-6, -24, −30, −31, and −34 are shown in purple.

*Vir-S309*: S309 (Dejnirattisai et al., 2021a; Pinto et al., 2020; Sun and Ho, 2020) retains some activity across the Omicron lineage, but notably less against BA.2. S309 binds the right flank with H3 contacting G339 and the N343 glycan, which is close to the serine 371, 373, and 375 mutations. 371 is a Phe in BA.2 compared to a Leu in BA.1 and superposition of the structure of BA.1 in complex with S309 (McCallum et al., 2022) on our BA.2 structure (see below) shows that the bulky Phe protrudes outwards disturbing the glycan attached to residue 343 of the RBD (Figure 4A). This sugar is critical for S309 binding, explaining the 126-fold reduction of neutralization titre to BA.2 compared to Victoria. Furthermore, neutralization of BA.1.1 is 4-fold worse than BA.1, due to the R346K mutation, since the shortened side chain cannot interact as effectively with Asp 93 of the S309 heavy chain (Figure 4B). Neutralization of BA.2 is approximately 20-fold worse than BA.1, consistent with SPR analysis which showed that binding to BA.2 is 15-fold weaker than to BA.1 (Figures S2H and S2I).

### Quantitative dissection of the nature of the Omicron mAb responses

We applied a neutralization-correlation method, which takes neutralization results for mAbs against various virus strains, calculates correlation coefficients for all possible pairs of mAbs, and then clusters the mAbs (Dejnirattisai et al., 2021a). Pseudovirus neutralization data (Figure 4C) for early pandemic (Dejnirattisai et al., 2021a), Beta (Liu et al., 2021b), and BA.1 antibodies revealed (Figure 4D, Video S1) clear differences between the three sets. The BA.1 antibodies are almost entirely separated from early pandemic mAbs, presumably by selection/somatic mutations. BA.1 antibodies are also largely distinguishable from Beta antibodies after clustering, but a subset of Beta antibodies (Beta-27, Beta-40, Betas-47-50, Betas-53-56, two of which belong to gene family IGHV1-69), share greater similarity with Omicron antibodies. Further cluster dissection of the Omicron antibodies (Figure 4E) segregates five that have a different neutralization profile due to drop-out against Delta (Omi-6, -24, −30, −31, −34); four of these are IGHV1-69.

Video S1. Antibody response correlation clustering, related to Figure 4D

### Fine mapping of RBD binding Omicron antibodies using competition measurements

**Figure 5:**
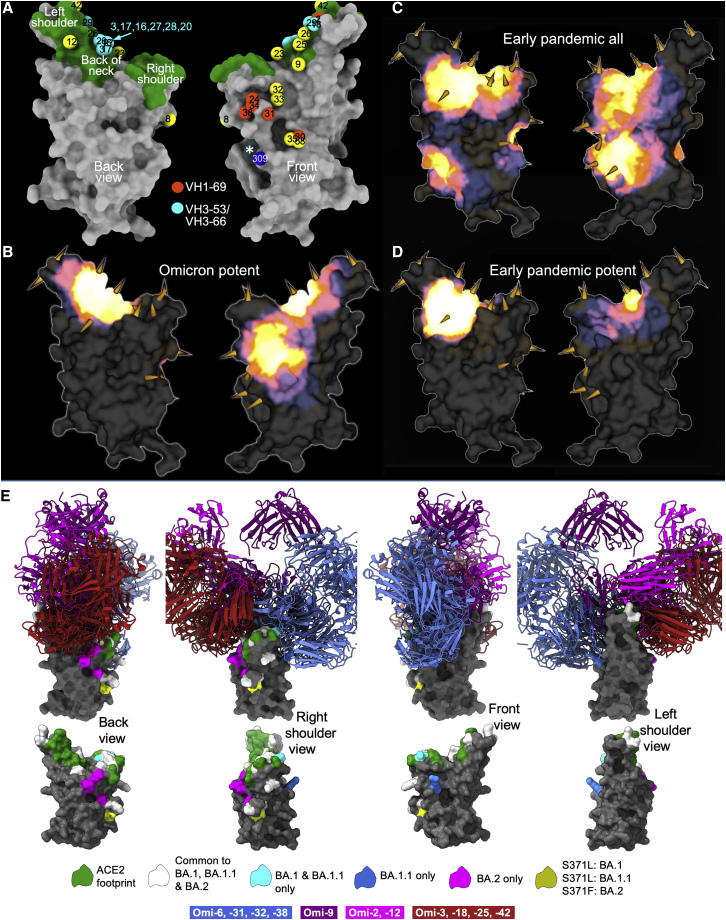
Omicron antibody mapping and structures of Omicron/Fab complexes (A) Mabscape antibody map (back and front views). Surface rendering of RBD (gray), ACE2 footprint in green, N343 glycan site in dark slate gray (marked with ^∗^). Spheres locate Omicron antibodies: IGHV3-53, cyan, IGHV1-69, orange-red, the rest in yellow; in addition, S309 is shown dark blue. (B) Heatmap of surface occupation of RBD by omicron antibodies (back and front views) by iron heat colors (black > blue > red > orange > yellow > white hot) according to the relative level of antibody contact, calculated for each surface vertex as the number of antibodies within a 10 Å radius. BA.1 mutations are shown by the spikes. (C) Heatmap, as in (B) but for the complete set of early pandemic response antibodies (Dejnirattisai et al., 2021a). (D) As (C) but showing only potent neutralizing antibodies. (C and D) are redrawn from (Dejnirattisai et al., 2022). (E) Superimposition based on the structures of the RBDs of 11 Omicron Fabs determined in complex with RBD or S (structure determination details in Table S3). The RBD surface for the Omi-3 complex is shown in gray. Residues in the ACE2 footprint and mutations associated with Omicron lineages are colored according to the key (as for Figure 1B). Fabs are are color-coded according to the site of interaction on the RBD. Front right shoulder binders in blue and back of the neck binders in red. Omi-2 and -12 are shown in magenta and Omi-9 in purple. The lower panel shows RBD alone orientated as in the upper panel. The four views correspond to successive 90° rotations about the vertical axis. See also Figures S2, S3, and S4 and Tables S3A and S3B.

Detailed 3D maps of the binding positions of antibodies can be obtained by combining competition data and some known antibody positions (Dejnirattisai et al., 2021a). We therefore performed pairwise biolayer interferometry (BLI) competition measurements on the 27 potent RBD-binding Omicron mAbs and several pre-pandemic mAbs of known binding position and obtained a map with average positional error of 9 Å (Figure 5A). The mAbs segregate into two principal clusters, which are a subset of the epitopes observed for the early pandemic virus and distinct from the focus seen for Beta (Figures 5A–5D) (Dejnirattisai et al., 2021a; Liu et al., 2021b).

The first antibody cluster includes the IGHV3-53 and IGHV3-66 type antibodies and is toward the back of the neck/left shoulder, extending up to the top of the left shoulder. This region corresponds to the major epitope for potent neutralizers in our early pandemic antibody panel (Figures 5B and 5D). Omi-9, which shows reduced neutralization of Beta and Gamma, positions close to residue 484, which is mutated from Glu to Lys in Beta/Gamma and to Ala in Omicron. The second, right shoulder, cluster was seen in the full set of early pandemic antibodies, above the S309 site (Figure 5A). This region is occupied by 5 of the 6 IGHV1-69 mAbs; the other, Omi-2, lies within the neck/left-shoulder cluster. IGHV1-69 mAbs Omi-24, 30, 31, and 34, which show reduced neutralization of Delta are placed close to residue 452, which is mutated from Leu to Arg in Delta. Omi-6, an IGHV4-4 antibody with reduced Delta neutralization (Figure 3A), occupies a similar position to the major cluster of IGHV1-69 antibodies.

### Structures of anti-Omicron Fab/RBD and Fab/spike complexes

**Figure 6:**
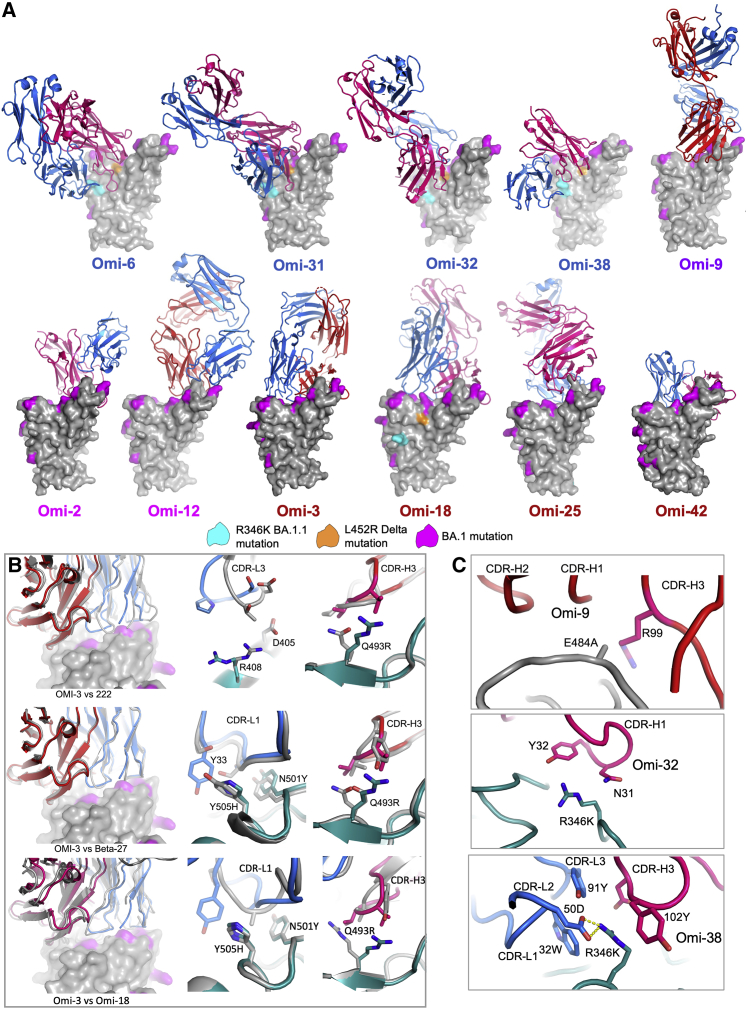
Structures of Omicron antibody complexes and correlation with sensitivity to RBD mutations (A) Representation similar to Figure 5E with approximate front view. The coloring scheme for RBD residues is shown in the key. Fab light chains (LC) are shown in blue and heavy chains (HC) in red. Label coloring follows the antibody coloring in Figure 5E. (B) IGHV3-53 adaptation. Front views of BA.1 RBD surface (BA.1 mutations in magenta) bound to Omi-3 Fab (HC red, LC blue). Top panel superimposed on with early pandemic mAb 222 complex (mAb 222 in gray). The right panels show the contacts with Omicron mutations with BA.1 RBD shown in green. The middle panel shows that the L3 loops pack differently against R408 and D405 (mutated to Ser and Asn respectively in BA.2). In the right panel, the H3 loop (red) and its contact with 493 are compared. The next row of panels below is as above for Omi3 vs. Beta-27 (Liu et al., 2021b). Note a Tyr in Omi-3 instead of a Ser in Beta-27 at residue 33 makes stacking contacts with H505. The bottom row of panels is the corresponding images for Omi-3 vs Omi-18. (C) Structural explanations for the relative sensitivity of Omi-9, -32 and -38 to mutations at spike residues 484 and 346. Note in Omi-9, the environment for residue 484 renders it sensitive to the E484K mutation found in Beta and Gamma, whilst Omi-32 and Omi-38 are knocked down and knocked out respectively by the mutation R346K. Omi-38 forms a salt bridge with LC 50D and hydrophobic interactions with H3 Tyr 103. See also Figures S3 and S4.

**Figure S3:**
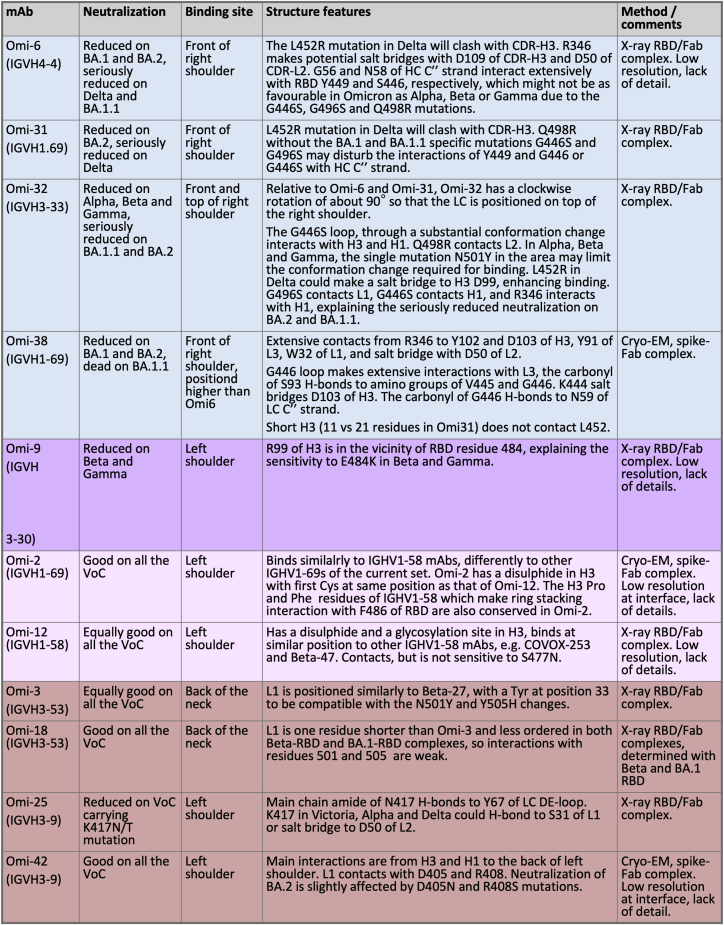
Summary structural analysis of Omicron elicited Fab complex structures Color coding matches that assigned to antibodies in Figure 5E. Related to Figures 5, 6, and 7.

**Figure S4:**
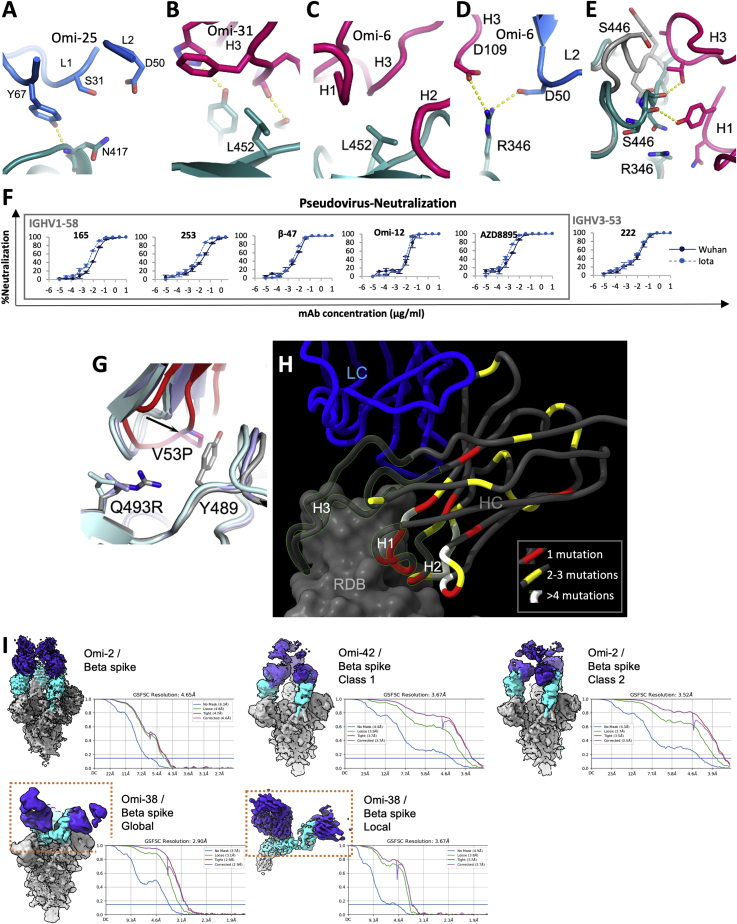
Antibody complex structures (A) Sensitivity of Omi-25 to K417 N/T. K417 can favourably interact with S31 and D50 in Victoria Alpha and Delta. (B and C) explain sensitivity to the Delta L452R mutation, since this residue lies just underneath the H3 loop in Oni-31 (B) and Omi-6 (C). The RBD is shown in green, the HS in red and LC in blue. (D) Sensitivity of Omi-6 to BA.1.1 through specific LC and HC interactions with R346. (E) Omi-32 causes large conformational changes in the G446S loop of the BA.1 RBD (shown in green) compared to the structure of the BA.1 RBD seen in the Omi-3 complex (gray). (F) pseudovirus neutralization curves for selected IGHV1-58 mAb and control IGHV3-53 mAb 222 against Wuhan and Iota (S477N). (G) The somatic mutation V53P contributes to re-folding of the H3 loop so that Q493R can be accommodated in Omi-12. (H) Somatic mutations in potent mAbs belonging to the IGHV1-69 gene family. Mutations are mapped onto Omi-2 (which has the longest H3 loop). Mutations are counted for the 6 antibodies listed in Table S1. Bound RBD is shown in gray, the mAb light chain in blue and the heavy chain in dark gray with somatic mutations colored according the frequency of changes from germline (dark gray to red to yellow to white, according to the key shown). The H1-3 loops are shown semi-transparent with a green outline. (I) cryo-EM maps for complexes of Omi-2, -38 and −42 with Beta S (shown in gray), RBD in cyan and Fab in purple. The relevant FSC plots are shown alongside each structure. The locally refined Omi-38 map is also shown, corresponding to the region boxed in the global map. Related to Figures 5, 6, and 7.

To further understand the basis of cross-reactivity and potency, we determined a number of structures by crystallography and cryo-EM (Figures 5E, 6, S3, S4A–S4E, and S4G–S4I; Tables S3 and S4), to give structural information on the binding of 11 of the 28 most potent antibodies, although for several the resolution was limited, and for some a structurally characterized nanobody (Huo et al., 2021) or Fab, or both (Zhou et al., 2020; Dejnirattisai et al. 2021a; Liu et al., 2021b), were required as crystallization chaperones. The binding sites show excellent agreement with those determined from the competition measurements, falling into two broad binding areas (Figures 5E and 6A). Table S3 summarizes the features observed.

### Back of the neck/left shoulder epitope binders

Omi-3 and -18 are representative of IGHV3-53 and IGHV3-66 antibodies that bind at the back of the neck and account for 9/28 of the most potent antibodies. They show how these antibodies can be adapted to broadly neutralize all major SARS-CoV-2 variants (Figure 6B). A problem for many IGHV3-53/66 antibodies is that most VoC harbor mutation N501Y, which introduces a steric clash with the LC CDR1 (L1) abrogating binding. However, we have previously reported two mechanisms for avoiding this clash (Dejnirattisai et al., 2021b; Liu et al., 2021b), by (1) mitigating the contact by inserting a Pro into the L1 loop or (2) shifting the L1 loop away from N501Y (Dejnirattisai et al., 2021b; Liu et al., 2021b). Omi-3 achieves resilience by repositioning the L1 loop in a mechanism similar to (2), whilst Omi-18 shortened the L1 loop, which becomes flexible enough to accommodate mutations at residues 501 and 505 (Figures 6B and S3).

We have determined structures for five mAbs within the neck/left shoulder cluster: Omi-2, -9, -12, −25, and −42. Some broadly neutralize all VoC, while others are sensitive to the mutations at residue 417 and 484 found in Beta and Gamma (explained for Omi-25 in Figure S4A). In terms of overall pose Omi-9 is an outlier, being perched upright on the RBD, whilst the others approach from the back (Figure 5E). Omi-2 belongs to the IGHV1-69 gene family but has features in common with Omi-12, the only member of the IGHV1-58 gene family found in the set of 28 potent antibodies. In particular, Omi-2 and Omi-12 have a disulphide bond and Pro and Phe residues at the same positions in the H3 loop which mediate interactions with F486 of the RBD; these commonalities appear to drive Omi-2 to adopt almost exactly the same pose as Omi-12, which differs from the other potent antibodies that bind in this region (Figure 6A). Note that while Omi-12, like many other IGHV1-58 antibodies, is glycosylated in the H3 loop, Omi-2 is non-glycosylated (Dejnirattisai et al., 2021a; Liu et al., 2021b).

### Front of right shoulder epitope binders

This cluster harbors all IGHV1-69 mAbs except Omi-2. As expected, these antibodies (structures obtained for Omi-31 and -38) attack the RBD from the front and sit above the binding site of Vir-S309. Changes, especially in the H3 loops, explain their differing specificities (Figure S3). Omi-6 and -32 bind at the same site, although Omi-6 binds a little lower and Omi-32 is rotated clockwise by ∼90° (Figure 6). The specific sensitivities of these antibodies to Delta and BA.1.1 is explained in Figures S3 and S4B–S4D. Omi-32 induces a large rearrangement in the 446 loop of the BA.1 RBD (Figure 4E). Omi-32 and -33 are clonally related and bind in the same way. Omi-33 showed 41-fold greater activity against BA.1.1 than Omi-32 (Figure 3B); this is because mutations in contact residues in L1 and H1 allow Omi-33 to better tolerate the change at 346 in BA.1.1. Antibodies binding at this epitope tend to be less broadly cross-reactive than those binding to the neck/left shoulder, due to a high concentration of mutations in the VoC, notably residues 346, 446, 452, 496, and 498.

### Example of RBD mutations repositioning an early pandemic mAb

Detectable residual activity for mAb 150 (IGHV3-53) was observed with BA.1, BA.1.1, and BA.2 (Table S2B). Structural analysis (Table S3) revealed binding to be broadly similar to that observed previously for early pandemic virus (Dejnirattisai et al., 2021a), although the Fab was translated by several Å and formed looser interactions, consistent with almost complete loss of neutralization activity.

### Effects of somatic mutation

In a set of potent early pandemic antibodies, the IGHV1-58 gene family was the second most highly represented (4/20) (Dejnirattisai et al., 2021a); however, they constitute only 1/28 in the Omicron set, and it is notable that other IGHV1-58 antibodies such as AZD8895 and representatives from our previous studies such as mAbs 55, 165, 253, and Beta-47 show large or complete loss of neutralization activity against Omicron BA.1 (Figures 3C and S4F) (Dejnirattisai et al., 2021a). The structural basis for the retention of activity of Omi-12 on BA.1 appears to be a somatic mutation in the HC CDR2 loop (V53P), which allows the RBD mutation Q493R to be accommodated (Figure S4G). Overall, we found higher levels of somatic mutation in both heavy and light chains of Omicron mAbs than in the early pandemic set of antibodies. Taking the IGHV1-69 gene family as an exemplar (Figure S4H), the changes are largely focused directly on the H2 and H1 loops, and residues adjacent to them in the sequence and in the 3D structure (notably the DE loop), with almost none at the interface with the LC.

### Structure of BA.2 RBD and ACE2 affinity

**Figure 7:**
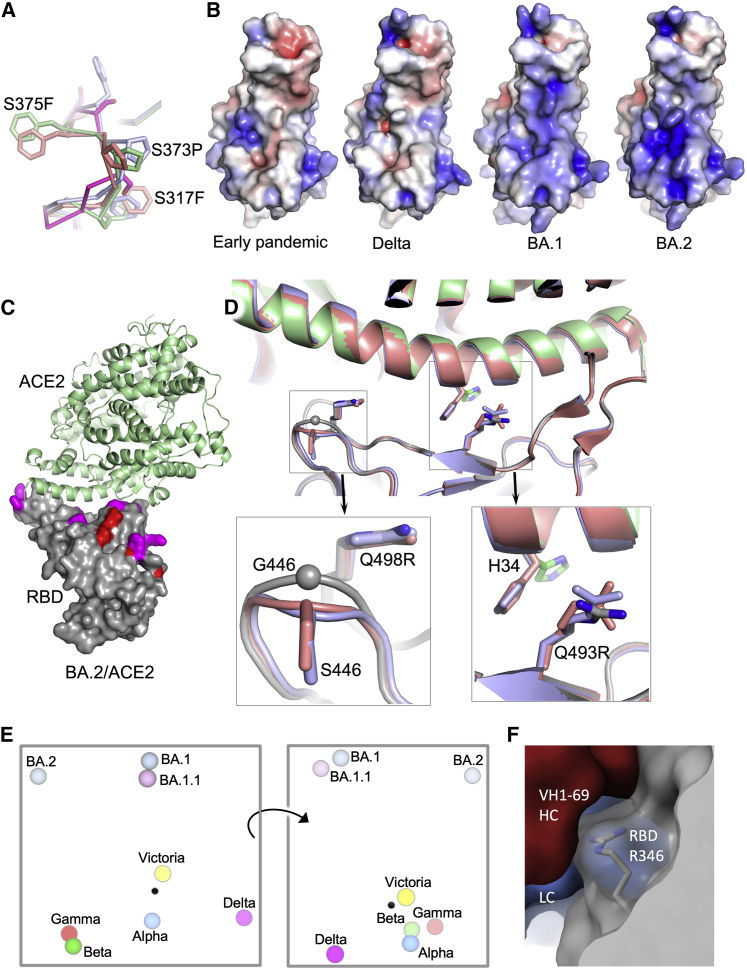
BA.2 RBD structure and ACE2 affinity (A) Residues 371–376 are seen in different conformations and compared with those of BA.1 RBD (bright red). (B) Electrostatic surfaces of the early pandemic, Delta, BA.1, and BA.2 RBDs. (C) Complex of ACE2 (green ribbons) and BA.2 RBD (gray surface with Omicron mutations colored). (D) Differences of ACE2 and BA.2 RBD interface with that of two previously reported ACE2/BA.1 RBD complexes (salmon and blue, PDB IDs 7TN0 and 7WB [Han et al., 2022; McCallum et al., 2022]). (E) Orthogonal views of the antigenic landscape for previous VoC and BA.1.1, BA.1, and BA.2, calculated from pseudovirus neutralization data. Distance between two positions is proportional to the reduction in neutralization titre when one of the corresponding strains is challenged with serum derived by infection by the other. (F) Front right shoulder binding IGHV1-69 Omi-38 (HC red, LC blue) contact with RBD R346 (gray). See also Figures S3 and S4 and Tables S3A and S3B.

We determined the structure of BA.2 RBD in complex with ACE2 (Table S3). As expected, the BA.2 RBD structure is very similar to that of BA.1 (Dejnirattisai et al., 2022; Han et al., 2022; McCallum et al., 2022). Although the three serine residues mutated in BA.1 RBD: S371L, S373P, and S375F are also mutated in BA.2, the mutation at 371 is to a Phe, representing a single codon mutation from early pandemic viruses, whereas the S371L mutation in BA.1 requires two mutations in the codon. BA.2 may therefore have features common to earlier versions of the Omicron lineage. The bulkier Phe protrudes from the structure in BA.2. In addition, the independent views provided by different crystal forms show that it adopts a range of conformations (Figure 7A), likely due to differing crystal contacts, reflecting flexibility in this loop region (also flexible in other variants). These changes may affect the presentation of the RBDs (Dejnirattisai et al., 2022).

We measured the affinity of BA.1 and BA.2 Spike and RBD for ACE2 by SPR (Figures S2J–S2O). The affinity of BA.2 RBD was slightly increased compared to early virus and BA.1 (∼2-fold, K_D_ = 4.0 nM), although affinities are similar among the three Spikes. The RBD binding probably gives the best indication of the intrinsic ACE2 affinity, and as reported earlier (Dejnirattisai et al., 2022), the affinity of RBD for BA.1 was on a par with that of the early virus, 7.8 nM and 7.3 nM, respectively (binding data for Omicron RBDs are shown in Figures S2A–S2L together with the binding of selected mAbs), implying that the increased affinity imparted by S477N, Q498R, and N501Y is counterbalanced by other mutations in the ACE2 footprint. Earlier measurements of the contributions of individual mutations to binding affinity (Dejnirattisai et al., 2022) show that G496S and the triple-mutation S371L, S373P, and S375F reduce binding by 2-fold and 2.2-fold, respectively, whereas BA.2 lacks G496S and has S371F. This may account for some of the difference, but more likely mutations on the edge of the ACE2 footprint (R408S and D405N only present in BA.2, G446S and G496S only present in BA.1) enhance binding of BA.2 to ACE2. This is confirmed by the structure of the BA.2/ACE2 complex (Figures 7B–7D; Table S3), which shows the same mode of engagement, with marginal additional binding conferred by improved charge complementarity with ACE2. Structural differences are observed at RBD residue G446 and at ACE2 H34 whose side chain has rotated ∼120° relative to the BA.1 RBD/ACE2 complex (Han et al., 2022; McCallum et al., 2022).

### The antigenic cartography of the Omicron sub-lineages

Using early pandemic, Alpha, Beta, Gamma, Delta, BA.1 sera together with vaccine sera in pseudoviral neutralization assays against Victoria, Alpha, Beta, Gamma, Delta, BA.1, BA.1.1, and BA.2, including some published data (Dejnirattisai et al., 2021a, 2021b, 2022; Liu et al., 2021a; Supasa et al., 2021; Zhou et al., 2021), we have extended the analysis recently reported for BA.1, modelling individual viruses independently and allowing for serum-specific scaling of the responses (Dejnirattisai et al., 2022). The measured and modeled responses are shown in Figure S2P (with 1,238 observations and 332 parameters, the residual error is 20.8%). The variant map is well described in three dimensions and presented in Video S2, with orthogonal projections shown in Figure 7E. Early pandemic, Alpha, Beta, Gamma, and Delta are roughly in a plane centered on the early pandemic virus. The Omicron sub-lineages are grouped together at a considerable distance from the earlier viral variants. BA.1 and BA.1.1 clustered very closely together, with BA.2 more distant.

Video S2. Antigenic cartography three-dimensional analysis, related to Figure 7E

## Discussion

The emergence of the highly transmissible Omicron variant and its extremely rapid global spread led to considerable concern; however, early data from South Africa that Omicron led to less severe disease has been borne out in waves of infection in other countries (Nealon and Cowling, 2022). Nevertheless, because of the very large number of infections, there remains considerable pressure on healthcare systems and significant numbers of deaths.

BA.1 and BA.2 were first reported at nearly the same time in November 2021. The BA.1 sub-lineage dominated the wave of Omicron infection in South Africa, but the proportion of Omicron infections caused by BA.2 has been increasing in several countries, and it is now dominant in Denmark, India, and the UK. It seems that BA.2 has a small transmission advantage over BA.1, and although there is no clinical evidence of increased disease severity, there is a suggestion from animal studies that this may be the case (Yamasoba et al., 2022). The sequence differences between these sub-lineages are likely to either alter the antigenicity of S such that reduced vaccine efficacy against BA.2 vs. BA.1 may be driving the transmission advantage, or alternatively may be increasing BA.2 receptor affinity. In line with this, we show a slight increase in the affinity of BA.2 RBD for ACE2 compared with BA.1 and a modest reduction in neutralization titers of BA.2 vs. BA.1 in vaccine serum, which is borne out in the antigenic cartography (Figure 7E; Video S2).

Following three doses of vaccine, particularly BNT162b2, good neutralizing titres of antibody against BA.1, BA.1.1, and BA.2 are induced, with only minor differences between them. Breakthrough Omicron infection in previously vaccinated individuals leads to an antibody response broadly effective against all VoC, including Omicron lineages. The similarity in neutralization titres suggests that reinfection of BA.1 exposed and vaccinated cases with BA.2 would be unlikely, at least in the short term; however, the concurrent high levels of infection by BA.1 and BA.2 have led to the identification of a BA.1/BA.2 recombinant virus XE (https://www.gov.uk/government/news/covid-19-variants-identified-in-the-uk). All the potent mAbs generated cross-neutralize Victoria, and many are broadly reactive against VoC. These responses may be recalled from memory B cells generated following vaccination, but since we do not have paired samples to analyze repertoire following vaccination before Omicron infection, this remains conjecture. It is noteworthy that vaccination, and in particular third dose vaccination, has been shown to induce a broader antibody response to VoC (Röltgen et al., 2022; Muecksch et al., 2022), targeting more conserved regions, than occurs following natural infection (https://doi.org/10.1016/j.cell.2022.01.018, PMID: 35194607).

Overall, the potent antibodies form two clusters (Figure 5B); the first, at the neck/left shoulder, includes antibodies that bind the back of the neck (e.g. IGHV3-53 antibodies), and those that bind more upright on the left shoulder (Omi-9); the second, on the front of the right shoulder is seen in the full set of our early pandemic antibodies, but does not include any of the highly potent antibodies in that set. Although most of the potent Omicron antibodies cross-neutralize all VoC, a subset shows poor or absent neutralization of Delta or Beta/Gamma. Omi-12, the most potent of the set of 28, belongs to the IGHV1-58 gene family, which has been isolated on several occasions following SARS-CoV-2 infection. It is anomalous in that it cross-neutralizes all VoC whilst other IGHV1-58 antibodies lose activity against BA.1, and this potency is recovered by somatic mutation.

The IGHV3-53 and IGHV3-66 families (9/27), form the most frequent public antibody response in the Omicron set and in the response to early pandemic virus (Dejnirattisai et al., 2021a; Yuan et al., 2020). Most early pandemic examples show reductions or loss of activity on 501Y containing VoCs, and we find that the appropriate length of H3 and L3 together with other changes in H3 can place L1 to accommodate 501Y and other mutations present in the Omicron lineage (Figure 6B).

The second most abundant IGHV family amongst the Omicron antibodies (6/27) was IGHV1-69, which also featured in a panel of potent mAbs isolated from Beta-infected cases (Liu et al., 2021b). We find that most of these bind in a similar way to the right shoulder, with several affected by the R346K mutation on BA.1.1, presumably due to stabilizing contacts analogous to that seen for S309 (Figure 7F). Interestingly the exception to this binding pattern is Omi-2 which binds in the other major cluster (Figure 6A).

Whilst the neutralization properties of most Omicron monoclonal antibodies isolated in this study did not show differences against BA.1, BA.1.1 and BA.2, some clinical mAbs showed differences, in particular REGN10987 regained some activity against BA.2 and AZD1061 regained most activity against BA.2 but lost activity against BA.1.1 compared to BA.1. Of particular concern S309, the activity of which is already reduced 6-fold against BA.1 (Dejnirattisai et al., 2022), was reduced a further 4-fold against BA.1.1 and a further 20-fold against BA.2. Although in the short term, genotyping may allow more efficient targeting of mAb therapy, there is a need to develop new Omicron specific antibodies to add to existing SARS-CoV-2 monoclonal antibody cocktails, or to develop broadly cross-reactive antibodies, to provide pre-exposure prophylaxis or post-exposure treatment to the many immunosuppressed patients unable to mount protective responses following vaccination.

In summary, we have presented a structure-function analysis of potent human antibodies induced by Omicron BA.1 breakthrough infection in SARS-CoV-2 vaccinated individuals. Many show broad activity against all VoC and may have been generated from vaccine memory responses. Overall, the structural studies demonstrate there is still space available on the RBD for the binding of potent mAbs able to broadly neutralize variants of concern. It also illustrates the extraordinary plasticity of the public antibody responses through IGHV3-53/66 and IGHV1-58 where neutralizing activity against BA.1 and other VoC can be restored by variation in CDR length and somatic mutation.

### Limitations of the study

Some limitations of this study are that as the neutralization assays are performed *in vitro*, they are not affected by antibody-dependent cellular cytotoxicity or complement-dependent cytotoxicity, which may augment the function of poorly neutralizing antibody *in vivo*. Furthermore, we have not studied the effects of the T cell response, which is known to withstand changes in the VoC more robustly than the antibody response and to persist, which may contribute to the protection from severe disease if the antibody response fails to block infection.

## STAR★Methods

### Key resources table

**Table:** 

REAGENT or RESOURCE	SOURCE	IDENTIFIER
**Antibodies**		

Fab	Dejnirattisai et al., (2021a)	N/A
IgG	Dejnirattisai et al., (2021a) and, (Liu et al., 2021b)	N/A
Human anti-NP (mAb 206)	Dejnirattisai et al., (2021a)	N/A
EY6A mAb	Zhou et al., (2020)	N/A
Regeneron mAbs	AstraZeneca	Cat#REGN10933, and REGN10987
AstraZeneca mAbs	AstraZeneca	Cat#AZD1061, AZD8895
Vir mAbs	Adagio	Cat#S309
Lilly mAbs	Adagio	Cat#Ly-CoV555, and Cat#Ly-CoV16
Adagio mAbs	Adagio	Cat#ADG10, Cat#ADG20, and Cat#ADG30
Anti-Human IgG (Fc specific)-Peroxidase	Sigma	Cat#A0170
Polyclonal Rabbit Anti-Goat Immunoglobulins/FITC	DAKO	Cat#F0250
Anti-*c*-Myc 9E10 antibody	Biolegend	Catt#626872
Anti-mouse IgG(Fc specific)-FITC antibody	Merck/Sigma Aldrich	Catt#F4143

**Bacterial and virus strains**		

SARS-CoV-2 (Australia/VIC01/2020)	Caly et al. (2020)	N/A
SARS-CoV-2/Alpha	Public Health England	N/A
SARS-CoV-2/Beta	Public Health England	N/A
SARS-CoV-2/Gamma	(Dejnirattisai et al., 2021b)	N/A
SARS-CoV-2/Delta	W. Barclay	Imperial College London
SARS-CoV-2/Omicron	This paper	N/A
SARS-CoV-2/B.1.525	Wendy Barclay and Thushan De-Silva	N/A
DH5α bacteria	In Vitrogen	Cat#18263012
*E. coli* cloni 10G cells	Lucigen, USA	Cat#60117–1
DH5α bacteria	Invitrogen	Cat# 18263012

**Biological samples**		

*Saccharomyces cerevisiae* EBY100	ATCC	Cat#MYA-4941
Serum from Pfizer-vaccinated individuals	University of Oxford	N/A
Serum from AstraZeneca-Oxford-vaccinated individuals	University of Oxford	N/A
PBMCs from SARS-CoV-2 patients	John Radcliffe Hospital in Oxford UK	N/A
Plasma from SARS-CoV-2 patients	John Radcliffe Hospital in Oxford UK, South Africa, and FIOCRUZ (WHO) Brazil	N/A

**Chemicals, peptides, and recombinant proteins**		

His-tagged SARS-CoV-2 RBD		
His-tagged SARS-CoV-2/Omicron RBD	This paper	N/A
His-tagged SARS-CoV-2 RBD-62	(Zahradnik et al., 2021)	N/A
His-tagged SARS-CoV-2 RBD N501Y	Supasa et al., (2021)	N/A
His-tagged SARS-CoV-2 RBD K417N, E484K, N501Y	Zhou et al., (2021)	N/A
His-tagged SARS-CoV-2 RBD K417T, E484K, N501Y	Dejnirattisai et al., (2021b)	N/A
His-tagged SARS-CoV-2 RBD L452R, T478K	Liu et al., (2021b)	N/A
His-tagged human ACE2	Liu et al. 2021a	N/A
Human ACE2-hIgG1Fc	Liu et al., 2021a	N/A
His-tagged 3C protease	Libby et al., 1988	N/A
Phosphate buffered saline tablets	Sigma-Aldrich	Cat#P4417
Dulbecco’s Modified Eagle Medium, high glucose	Sigma-Aldrich	Cat#D5796
Dulbecco’s Modified Eagle Medium, low glucose	Sigma-Aldrich	Cat#D6046
FreeStyle™ 293 Expression Medium	Gibco	Cat#12338018
L-Glutamine–Penicillin–Streptomycin solution	Sigma-Aldrich	Cat#G1146
GlutaMAX™ Supplement	Gibco	Cat#35050061
UltraDOMA PF Protein-free Medium	Lonza	Cat#12-727F
Opti-MEM™	Gibco	Cat#11058021
Fetal Bovine Serum	Gibco	Cat#12676029
Polyethylenimine, branched	Sigma-Aldrich	Cat#408727
Carboxymethyl cellulose	Sigma	Cat#C4888
Strep-Tactin®XT	IBA Lifesciences	Cat#2-1206-025
HEPES	Melford	Cat#34587-39108
Sodium Chloride	Honeywell	Cat#SZBF3340H
LB broth	Fisher Scientific UK	Cat#51577-51656
Mem Neaa (100X)	Gibco	Cat#2203945
Trypsin-EDTA	Gibco	Cat#2259288
TrypLE™ Express Enzyme	Gibco	Cat#12604013
L-Glutamine 200 mM (100X)	Gibco	Cat#2036885
SYPROorange (5000X in DMSO)	Thermo	Cat#S6651
Isopropyl β-d-1-thiogalactopyranoside	Meridian Bioscience	Cat#BIO-37036
Kanamycin	Melford	Cat#K22000
Lysozyme	Sigma-Aldrich	Cat#L6876
Tris-base	Melford	Cat#T60040
Imidazole	Sigma-Aldrich	Cat#56750
Triton-X-100	Sigma-Aldrich	Cat#8787
Turbonuclease	Sigma-Aldrich	Cat#T4330
RNAse A	Qiagen	Cat#158922
NaCl	Sigma-Aldrich	Cat#S9888
MgSO4	Sigma-Aldrich	Cat#746452
Na2HPO4	Melford	Cat#S23100
NaH2PO4	Melford	Cat#S23185
SD-CAA media	(Zahradnik et al., 2021)	N/A
CF640-ACE2	(Zahradnik et al., 2021)	N/A
HBS-EP+ Buffer 10×	Cytiva	Cat# BR100669
Regeneration Solution (glycine-HCl pH 1.7)	Cytiva	Cat# BR100838
Sensor Chip Protein A	Cytiva	Cat#29127555
His-tagged SARS-CoV-2 BA.1 variant RBD	This paper	N/A
His-tagged SARS-CoV-2 BA.2 variant RBD	This paper	N/A
SARS-CoV-2 BA.1 variant Spike	This paper	N/A
SARS-CoV-2 BA.2 variant Spike	This paper	N/A
Streptavidin-APC	Biolegend	Cat# 405207
Streptavidin-APC	Biolegend	Cat# 405207
RNase inhibitor	Promega	Cat# N2611
Protein G Plus/Protein A Agarose	Millipore	Cat#IP10
Pierce™ Fab Preparation Kit	Thermo Fisher	Cat#44985
Twin-Strep-tag® Capture Kit	IBA-Lifesciences	Cat# 2-4370-000
PEGRx 2	Hampton Research	HR2-084
ProPlex™ HT-96	Molecular Dimensions	MD1-42
JCSG-plus™ HT-96	Molecular Dimensions	MD1-40

**Critical commercial assays**		

Bright-Glo Luciferase Assay System	Promega	Cat# E2620
HIV Type 1 p24 Antigen ELISA 2.0	ZeptoMetrix	Cat# 0801002

**Deposited data**		

Crystal structure of SARS-CoV-2 BA.1-RBD/Omi-3 and EY6A Fab complex	This paper	PDB: 7ZF3
Crystal structure of SARS-CoV-2 BA.1-RBD/Omi-9 Fab and NbF2 complex	This paper	PDB: 7ZF4
Crystal structure of SARS-CoV-2 BA.1-RBD/Omi-12 and Beta-54 Fab complex	This paper	PDB: 7ZF5
Crystal structure of Omi-12 Fab	This paper	PDB: 7ZF6
Crystal structure of SARS-CoV-2 BA.2-RBD/ACE2 complex	This paper	PDB: 7ZF7
Crystal structure of SARS-CoV-2 BA.2-RBD/COVOX 150 Fab complex	This paper	PDB: 7ZF8, PDB:7ZF9
Crystal structure of BA.1-RBD/Omi-18 and Omi-31 Fab and NbC1complex	This paper	PDB: 7ZFB
Crystal structure of SARS-CoV-2 BA.1-RBD/Omi-32 Fab and NbC1 complex	This paper	PDB: 7ZFE
Crystal structure of SARS-CoV-2 Beta-RBD/Omi-18 and Omi31 Fab and NbC1 complex	This paper	PDB: 7ZFC
Crystal structure of Omi-42 Fab	This paper	PDB: 7ZFF
Crystal structure of SARS-CoV-2 BA.1-RBD/Omi-25 Fab complex	This paper	PDB: 7ZFD
CryoEM structure of Omi-2 Fab in complex with SARS-CoV-2 Beta Spike ectodomain	This paper	EMD-14887, PDB:7ZR9
CryoEM structure of Omi-38 Fab in complex with SARS-CoV-2 Beta Spike ectodomain	This paper	EMD-14910, PDB:7ZRC
CryoEM structure of Omi-38 Fab in complex with SARS-CoV-2 Beta Spike RBD (locally refined)	This paper	EMD-14886, PDB: 7ZR8
CryoEM structure of Omi-42 Fab in complex with SARS-CoV-2 Beta Spike ectodomain	This paper	EMD-14885, PDB: 7ZR7

**Experimental models: Cell lines**		

HEK293S GnTI- cells	ATCC	Cat#CRL-3022
HEK293 cells	ATCC	Cat#CRL-3216
Expi293F™ Cells	Gibco,	Cat#A14527
HEK293T/17 cells	ATCC	Cat#CRL-11268™
HEK293T cells	ATCC	Cat#CRL-11268
Hamster: ExpiCHO cells	Thermo Fisher	Cat#A29133
Vero CCL-81 cells	ATCC	Cat#CCL-81
VeroE6/TMPRSS2 cells	NIBSC	Ref. no. 100978

**Recombinant DNA**		

Vector: pHLsec	Aricescu et al., 2006	N/A
Vector: pNEO	Aricescu et al., 2006	N/A
Vector: pHLsec-SARS-CoV-2 spike of BA.1	This paper	N/A
Vector: pTTGneO-SARS-CoV-2 spike of BA.2	This paper	N/A
Vector: pTTGneO-SARS-CoV-2 RBD of BA.2	This paper	N/A
Vector: pNEO-SARS-CoV-2 RBD of BA.1	This paper	N/A
Vector: pCMV-VSV-G	Stewart et al., 2003	Addgene plasmid # 8454
pHR-SIN-ACE2	Alain Townsend	N/A
Vector: pOPING-ET	Nettleship et al., 2008	N/A
Vector: human IgG1 heavy chain	German Cancer Research Center, Heidelberg, Germany (H. Wardemann	N/A
Vector: human lambda light chain	German Cancer Research Center, Heidelberg, Germany (H. Wardemann	N/A
Vector: human kappa light chain	German Cancer Research Center, Heidelberg, Germany (H. Wardemann	N/A
Vector: Human Fab	Univeristy of Oxford	N/A
Vector: pJYDC1	Adgene	ID: 162458
Vector: p8.91	Di Genova et al., 2021	Nigel Temperton
Vector: pCSFLW	Di Genova et al., 2021	Nigel Temperton
TM149 BirA pDisplay	University of Oxford, NDM (C. Siebold)	N/A

**Software and algorithms**		

COOT	Emsley and Cowtan, 2004	https://www2.mrc-lmb.cam.ac.uk/personal/pemsley/coot/
Xia2-dials	Winter et al. (2018)	https://xia2.github.io/index.html
PHENIX	Liebschner et al. (2019)	https://www.phenix-online.org/
PyMOL	Warren DeLano and Sarina Bromberg	https://pymol.org/
Data Acquisition Software 11.1.0.11	Fortebio	https://www.fortebio.com/products/octet-systems-software
Data Analysis Software HT 11.1.0.25	Fortebio	https://www.fortebio.com/products/octet-systems-software
Prism 9.0	GraphPad	https://www.graphpad.com/scientific-software/prism/
CryoSPARC v2.15.1-live	Structura Biotechnology Inc.	https://cryosparc.com/
SerialEM (version 3.8.0 beta)	https://bio3d.colorado.edu/SerialEM/; (Mastronarde, 2005)	N/A
EPU	Thermo Fisher	https://www.thermofisher.com/uk/en/home/electron-microscopy/products/software-em-3d-vis/epu-software.html
IBM SPSS Software 27	IBM	https://www.ibm.com
mabscape	This paper	https://github.com/helenginn/mabscape https://snapcraft.io/mabscape
Biacore T200 Evaluation Software 3.1	Cytiva	www.cytivalifesciences.com
Flowjo 10.7.1	BD	https://www.flowjo.com
SnapGene software 5.3.2	Insightful Science	www.snapgene.com

**Other**		

X-ray data were collected at beamlines I03 and I04, Diamond Light Source, under proposal lb27009 for COVID-19 rapid access	This paper	https://www.diamond.ac.uk/covid-19/for-scientists/rapid-access.html
TALON® Superflow Metal Affinity Resin	Clontech	Cat#635668
HiLoad® 16/600 Superdex® 200 pg	Cytiva	Cat#28-9893-35
Superdex 200 increase 10/300 GL column	Cytiva	Cat#28990944
HisTrap nickel HP 5-mL column	Cytiva	Cat#17524802
HiTrap Heparin HT 5-mL column	Cytiva	Cat#17040703
Amine Reactive Second-Generation (AR2G) Biosensors	Fortebio	Cat#18-5092
Octet RED96e	Fortebio	https://www.fortebio.com/products/label-free-bli-detection/8-channel-octet-systems
Buffer exchange system “QuixStand”	GE Healthcare	Cat#56-4107-78
Cartesian dispensing system	Genomic solutions	Cat#MIC4000
Hydra-96	Robbins Scientific	Cat#Hydra-96
96-well crystallization plate	Greiner bio-one	Cat#E20113NN
Crystallization Imaging System	Formulatrix	Cat#RI-1000
Sonics vibra-cell vcx500 sonicator	VWR	Cat#432-0137
Cryo-EM data were collected at COSMIC, University of Oxford.	This paper	https://www.research-facilities.ox.ac.uk/view:facility/cosmic-cryo-em-facility
Cryo-EM data were collected at OPIC, Division of Structural Biology, University of Oxford	This paper	https://www.opic.ox.ac.uk/
Biacore T200	Cytiva	https://www.cytivalifesciences.com/en/us/shop/protein-analysis/spr-label-free-analysis/systems/biacore-t200-p-05644
QuixStand	GE Healthcare	Cat# 56-4107-78

### Resource availability

#### Lead contact

Resources, reagents and further information requirement should be forwarded to and will be responded by the lead contact, David I. Stuart (dave@strubi.ox.ac.uk).

#### Materials availability

Reagents generated in this study are available from the lead contact with a completed Materials Transfer Agreement.

### Experimental model and subject details

#### Study subjects

Monoclonal antibodies were isolated from individuals with sequence-confirmed Omicron infection in the early phase of the variant wave in late-2021. Following informed consent, individuals with omicron were co-enrolled into the ISARIC/WHO Clinical Characterisation Protocol for Severe Emerging Infections [Oxford REC C, reference 13/SC/0149] and the “Innate and adaptive immunity against SARS-CoV-2 in healthcare worker family and household members” protocol affiliated to the Gastro-intestinal illness in Oxford: COVID sub study [Sheffield REC, reference: 16/YH/0247] further approved by the University of Oxford Central University Research Ethics Committee. Diagnosis was confirmed through reporting of symptoms consistent with COVID-19 or a positive contact of a known Omicron case, and a test positive for SARS-CoV-2 using reverse transcriptase polymerase chain reaction (RT-PCR) from an upper respiratory tract (nose/throat) swab tested in accredited laboratories and lineage sequence confirmed through national reference laboratories. A blood sample was taken following consent at least 14 days after PCR test confirmation. Clinical information including severity of disease (mild, severe or critical infection according to recommendations from the World Health Organisation) and times between symptom onset and sampling and age of participant was captured for all individuals at the time of sampling.

#### Viral stocks

SARS-CoV-2/human/AUS/VIC01/2020(Caly et al., 2020), Alpha and Beta were provided by Public Health England, Gamma cultured from a throat swab from Brazil, Delta was a gift from Wendy Barclay and Thushan de-Silva, from the UK G2P genotype to phenotype consortium and Omicron was grown from a positive throat swab (IRAS Project ID: 269573, Ethics Ref: 19/NW/0730. Briefly, VeroE6/TMPRSS2 cells (NIBSC) were maintained in Dulbecco’s Modified Eagle Medium (DMEM) high glucose supplemented with 1% fetal bovine serum, 2mM Glutamax, 100 IU/mL penicillin-streptomycin and 2.5ug/mL amphotericin B, at 37°C in the presence of 5% CO2 before inoculation with 200ul of swab fluid. Cells were further maintained at 37 °C with daily observations for cytopathic effect (CPE). Virus containing supernatant were clarified at 80% CPE by centrifugation at 3,000 r.p.m. at 4°C before being stored at −80°C in single-use aliquots. Viral titers were determined by a focus-forming assay on Vero CCL-81 cells (ATCC). Sequencing of the Omicron BA.1 isolate shows the expected consensus S gene changes (A67V, Δ69–70, T95I, G142D/Δ143-145, Δ211/L212I, ins214EPE, G339D, S371L, S373P, S375F, K417N, N440K, G446S, S477N, T478K, E484A, Q493R, G496S, Q498R, N501Y, Y505H, T547K, D614G, H655Y, N679K, P681H, N764K, D796Y, N856K, Q954H, N969K, L981F), an intact furin cleavage site and a single additional mutation A701V. Sequencing of the BA.1.1 isolate shows an additional mutation R346K and lack of mutation A701V compared with BA.1, and sequencing of BA.2 confirmed the expected changes in the S gene (T19I, Δ24–26, A27S, G142D, V213G, G339D, S371F, S373P, S375F, T376A, D405N, R408S, K417N, N440K, S477N, T478K, E484A, Q493R, Q498R, N501Y, Y505H, D614G, H655Y, N679K, P681H, N764K, D796Y, Q954H and N969K). BA.1, BA.1.1 and BA.2 isolates have been fully sequenced and the deposited reads have INSDC accession numbers ERR8959182, ERR9321875 and ERR9321876 respectively. Cells were infected with the SARS-CoV-2 virus using an MOI of 0.0001.

Virus containing supernatant were harvested at 80% CPE and spun at 3000 rpm at 4°C before storage at −80°C. Viral titers were determined by a focus-forming assay on Vero cells. Victoria passage 5, Alpha passage 2 and Beta passage 4 stocks Gamma passage 1, Delta passage 3, BA.1 passage 2, BA.1.1 passage 2, and BA.2 passage 2 were sequenced to verify that they contained the expected spike protein sequence and no changes to the furin cleavage sites.

#### Bacterial strains and cell culture

Vero (ATCC CCL-81) and VeroE6/TMPRSS2 cells were cultured at 37°C in Dulbecco’s Modified Eagle medium (DMEM) high glucose (Sigma-Aldrich) supplemented with 10% fetal bovine serum (FBS), 2 mM GlutaMAX (Gibco, 35050061) and 100 U/mL of penicillin–streptomycin. HEK293T (ATCC CRL-11268) cells were passaged in DMEM high glucose (Sigma-Aldrich) supplemented with 10% FBS, 1% 100X Mem Neaa (Gibco) and 1% 100X L-Glutamine (Gibco) at 37°C with 5% CO_2_. To express Wuhan RBD, beta-RBD and ACE2, HEK293T cells were cultured in DMEM high glucose (Sigma) supplemented with 2% FBS, 1% 100X Mem Neaa and 1% 100X L-Glutamine at 37°C for transfection. Spike and Human mAbs were also expressed in HEK293T (ATCC CRL-11268) cells cultured in FreeStyle 293 Expression Medium (ThermoFisher, 12338018) at 37°C with 5% CO_2_. BA.1 and BA.2 RBDs were expressed in Expi293F™ Cells (ThermoFisher), cultured in FreeStyle™ 293 Expression Medium (ThermoFisher) at 30°C with 8% CO_2_. *E.coli DH5α* and Turbo Competent *E. coli* (NEB) bacteria were used for transformation and large-scale preparation of plasmids. Single colonies were picked and cultured in LB broth at 37°C at 200 rpm in a shaker overnight.

#### Sera from Pfizer vaccinees

Pfizer vaccine serum was obtained from volunteers who had received either one or two doses of the BNT162b2 vaccine. Vaccinees were Health Care Workers, based at Oxford University Hospitals NHS Foundation Trust, not known to have prior infection with SARS-CoV-2 and were enrolled in the OPTIC Study as part of the Oxford Translational Gastrointestinal Unit GI Biobank Study 16/YH/0247 [research ethics committee (REC) at Yorkshire & The Humber – Sheffield] which has been amended for this purpose on 8 June 2020. The study was conducted according to the principles of the Declaration of Helsinki (2008) and the International Conference on Harmonization (ICH) Good Clinical Practice (GCP) guidelines. Written informed consent was obtained for all participants enrolled in the study. Participants were studied after receiving two doses of, and were sampled approximately 28 days (range 25–38), after receiving two doses of Pfizer/BioNtech BNT162b2 mRNA Vaccine, 30 micrograms, administered intramuscularly after dilution (0.3 mL each), 17–28 days apart, then approximately 28 days (range 25–56) after receiving a third “booster dose of BNT162B2 vaccine. The mean age of vaccinees was 37 years (range 22–66), 21 male and 35 female.

#### AstraZeneca-Oxford vaccine study procedures and sample processing

Full details of the randomized controlled trial of ChAdOx1 nCoV-19 (AZD1222), were previously published (PMID: 33220855/PMID: 32702298). These studies were registered at ISRCTN (15281137 and 89951424) and ClinicalTrials.gov (NCT04324606 and NCT04400838). Written informed consent was obtained from all participants, and the trial is being done in accordance with the principles of the Declaration of Helsinki and Good Clinical Practice. The studies were sponsored by the University of Oxford (Oxford, UK) and approval obtained from a national ethics committee (South Central Berkshire Research Ethics Committee, reference 20/SC/0145 and 20/SC/0179) and a regulatory agency in the United Kingdom (the Medicines and Healthcare Products Regulatory Agency). An independent DSMB reviewed all interim safety reports. A copy of the protocols was included in previous publications (Folegatti et al., 2020).

Data from vaccinated volunteers who received two or three doses: Vaccine doses were either 5 × 10^10^ viral particles (standard dose; SD/SD cohort n = 21) or half dose as their first dose (low dose) and a standard dose as their second dose (LD/SD cohort n = 4). The interval between first and second dose was in the range of 8–14 weeks. Blood samples were collected and serum separated on the day of vaccination and on pre-specified days after vaccination e.g. 14 and 28 days after boost.

### Method details

#### Isolation of Omicron S-specific single B cells by FACS

Omicron S-specific single B cell sorting was performed as previously described (Dejnirattisai et al., 2021a). Briefly, PBMC were stained with LIVE/DEAD Fixable Aqua dye (Invitrogen) followed by recombinant trimeric S-twin-Strep of BA.1. Cells were then incubated with CD3-FITC, CD14-FITC, CD16-FITC, CD56-FITC, IgM-FITC, IgA-FITC, IgD-FITC, IgG-BV786 and CD19-BUV395, along with Strep-MAB-DY549 to stain the twin strep tag of the S protein. IgG+ memory B cells were gated as CD19^+^, IgG+, CD3^−^, CD14^−^, CD56^−^, CD16^−^, IgM-, IgA- and IgD-, and S+ was further selected and single cells were sorted into 96-well PCR plates with 10 μL of catching buffer (Tris, Nuclease-free-H2O and RNase inhibitor). Plates were briefly centrifuged at 2000ⅹg for 1 min and left on dry ice before being stored at −80°C.

#### Cloning and expression of Omicron S-specific human mAbs

Omicron S-specific human mAbs were cloned and expressed as described previously (Dejnirattisai et al., 2021a). Briefly, genes for Ig IGHV, Ig Vκ and Ig Vλ were recovered from positive wells by RT-PCR. Genes encoding Ig IGHV, Ig Vκ and Ig Vλ were then amplified using Nested-PCR by a cocktail of primers specific to human IgG. PCR products of HC and LCs were ligated into the expression vectors of human IgG1 or immunoglobulin κ-chain or λ-chain by Gibson assembly (Gibson, 2011). For mAb expression, plasmids encoding HCs and LCs were co-transfected by PEI-transfection into a HEK293T cell line, and supernatants containing mAbs were collected and filtered 4–5 days after transfection, and the supernatants were further characterized or purified.

#### ACE2 binding inhibition assay by ELISA

MAXISORP immunoplates were coated with 5 μg/mL of purified ACE2-His protein overnight at 4°C and then blocked by 2% BSA in PBS. Meanwhile, mAbs were serially diluted and mixed with 2.5 μg/mL of recombinant BA.1 trimeric S-twin-Strep. Antibody-S protein mixtures were incubated at 37 °C for 1 h. After incubation, the mixtures were transferred into the ACE2-coated plates and incubated for 1 h at 37°C. After wash, StrepMAB-Classic (2-1507-001, iba) was diluted at 0.2 μg/mL by 2% BSA and used as primary antibody followed by Goat anti-mouse IgG-AP (#A16093, Invitrogen) at 1:2000 dilution. The reaction was developed by adding PNPP substrate and stopped with NaOH. The absorbance was measured at 405nm. The ACE2/S binding inhibition was calculated by comparing to the antibody-free control well. IC50 was determined using the Probit program from the SPSS package.

#### Focus reduction neutralization assay (FRNT)

The neutralization potential of Ab was measured using a Focus Reduction Neutralization Test (FRNT), where the reduction in the number of the infected foci is compared to a negative control well without antibody. Briefly, serially diluted Ab or plasma was mixed with SARS-CoV-2 strains and incubated for 1 h at 37°C. The mixtures were then transferred to 96-well, cell culture-treated, flat-bottom microplates containing confluent Vero cell monolayers in duplicate and incubated for a further 2 h followed by the addition of 1.5% semi-solid carboxymethyl cellulose (CMC) overlay medium to each well to limit virus diffusion. A focus forming assay was then performed by staining Vero cells with human anti-NP mAb (mAb206) followed by peroxidase-conjugated goat anti-human IgG (A0170; Sigma). Finally, the foci (infected cells) approximately 100 per well in the absence of antibodies, were visualized by adding TrueBlue Peroxidase Substrate. Virus-infected cell foci were counted on the classic AID EliSpot reader using AID ELISpot software. The percentage of focus reduction was calculated and IC_50_ was determined using the probit program from the SPSS package.

#### Plasmid construction and pseudotyped lentiviral particles production

Pseudotyped lentivirus expressing SARS-CoV-2 S proteins were constructed as described before (Nie et al., 2020; Liu et al., 2021a; 2021b), with some modifications. Compared to Wuhan sequence, the gene sequences were designed to encode S protein of BA.1 (A67V, Δ69–70, T95I, G142D/Δ143-145, Δ211/L212I, ins214EPE, G339D, S371L, S373P, S375F, K417N, N440K, G446S, S477N, T478K, E484A, Q493R, G496S, Q498R, N501Y, Y505H, T547K, D614G, H655Y, N679K, P681H, N764K, D796Y, N856K, Q954H, N969K and L981F), BA.1.1 (BA.1 as above plus R346K), BA.2 (T19I, LPPA24S, G142D, V213G, G339D, S371F, S373P, S375F, T376A, D405N, R408S, K417N, N440K, S477N, T478K, E484A, Q493R, Q498R, N501Y, Y505H, D614G, H655Y, N679K, P681H, N764K, D796Y, Q954H and N969K. Briefly, synthetic codon-optimized SARS-CoV-2 BA.1 and BA.2 were custom synthesized by GeneArt (Thermo Fisher Scientific GENEART). The insert fragments and pcDNA3.1 vector were cloned using Gibson assembly. The Victoria (S247R) construct is as previously described in Liu et al. 2021a, 2021b.

To construct BA.1.1, mutagenic primers of R346K (R346K_F 5′-GTGTTCAATGCCACCAAATTCGCCAGCGTGTAC-3′ and R346K_R 5′-GTACACGCTGGCGAATTTGGTGGCATTGAACAC-3′) were PCR amplified by using BA.1 construct as a template, together with two primers of pcDNA3.1 vector (pcDNA3.1_BamHI_F 5′-GGATCCATGTTCCTGCTGACCACCAAGAG-3′ and pcDNA3.1_Tag_S_EcoRI_R 5′-GAATTCTCACTTCTCGAACTGAGGGTGGC-3′). Amplified DNA fragments were purified by using QIAquick Gel Extraction Kit (QIAGEN) and joined with pcDNA3.1 vector followed by Gibson assembly. All constructs were verified by Sanger sequencing after plasmid isolation using QIAGEN Miniprep kit (QIAGEN).

#### Pseudoviral neutralization test

The details of pseudoviral neutralization test were described previously (Liu et al. 2021a, 2021b) with some modifications. Briefly, neutralizing activity of potent monoclonal antibodies (mAbs) generated from donors who had recovered from Omicron- and Beta-infection as well as those who were infected during the early pandemic in UK were performed against Victoria, Omicron-BA.1, BA.1.1 and BA.2. A four-fold serial dilution of each mAb was incubated with pseudoviral particles at 37°C, 5% CO2 for 1 h. The stable HEK293T/17 cells expressing human ACE2 were then added to the mixture at 1.5 x 10^4^ cells/well. At 48 h post transduction, culture supernatants were removed and 50 μL of 1:2 Bright-GloTM Luciferase assay system (Promega, USA) in 1x PBS was added to each well. The reaction was incubated at room temperature for 5 min and the firefly luciferase activity was measured using CLARIOstar® (BMG Labtech, Ortenberg, Germany). The percentage of neutralization was calculated relative to the control. Probit analysis was used to estimate the value of dilution that inhibits half of the maximum pseudotyped lentivirus infection (PVNT50).

To determine the neutralizing activity of convalescent plasma/serum samples or vaccine sera, 3-fold serial dilutions of samples were incubated with the pseudoviral particles for 1 h and the same strategy as mAb was applied.

#### Antibody clustering on neutralization tests

Monoclonal antibodies isolated from patients during the early pandemic, Beta patients and Omicron patients along with a panel of neutralization titers against Victoria, Alpha, Beta, Gamma, Delta and Omicron-BA.1 pseudoviruses were clustered using cluster4x (Ginn, 2020). Neutralization titers >10 mg/ul were given a fixed value of 100 mg/ul and all neutralization values passed to cluster4x as log values.

#### Antigenic landscape mapping

Antigenic mapping was carried out as previously described (Dejnirattisai et al., 2022; Liu et al., 2021a). In short, each virus/vaccine was assigned a three-dimensional location. These were refined such that the distance between each virus (or vaccine) pair is proportional to the fall-off in neutralization capacity when a patient is infected/inoculated with one of the pair and their serum is challenged by the other. This used a panel of data derived from the following serum: Victoria, Alpha, Beta, Gamma, Delta, Omicron, Chadox-vaccinated (2x, 3x) 28 days after vaccination, Pfizer-vaccinated (2x, 3x) 28 days after vaccination. Neutralization titers were carried out against Victoria, Alpha, Beta, Gamma, Delta, BA.1, BA1.1 and BA.2 pseudoviruses (see Figure S2B for a full representation of collected data).

#### DNA manipulations

Cloning was done by using a restriction-free approach (Peleg and Unger, 2014). Mutagenic megaprimers were PCR amplified (KAPA HiFi HotStart ReadyMix, Roche, Switzerland, cat. KK3605), purified by using NucleoSpin® Gel and PCR Clean-up kit (Nacherey-Nagel, Germany, REF 740609.50) and cloned into pJYDC1 (Adgene ID: 162458) (Zahradnik et al., 2021). Parental pJYDC1 molecules were cleaved by *Dpn*I treatment (1 h, NEB, USA, cat. R0176) and the reaction mixture was electroporated into E.coli Cloni® 10G cells (Lucigen, USA). The correctness of mutagenesis was verified by sequencing.

#### Cloning of spike and RBD

Expression plasmids encoding Omicron spikes were constructed with human codon-optimized sequences from BA.1 (EPI_ISL_6640917) and BA.2 (EPI_ISL_6795834.2). The constructs of Wild-type and BA.1 Spike plasmids are the same as previously described (Dejnirattisai et al., 2021a). The gene of BA.1 RBD (319–541) was amplified using primers (5′-GCGTAGCTGAAACCGGCagagtgcagcctaccgagagc-3′ and 5′- gtcattcagCAAGCTttattagtgatggtgatggtgatgGAAATTCACGCACTTATTC-3′); BA.1 and BA.2 RBD (330–532) was amplified using primers (5′-GCGTAGCTGAAACCGGCcctaatatcaccaatctgtgc-3′ and 5′- gtcattcagCAAGCTttattagtgatggtgatggtgatgATTGGTGCTCTTCTTAGGGCC-3′); and the gene fragments were cloned into the pOPOINTTGneo vector as previously described (Huo et al., 2021). The construct was verified by Sanger sequencing.

#### Protein production

Protein expression and purification were conducted largely as described previously (Dejnirattisai et al., 2021a; Zhou et al., 2021). Twin-strep tagged Omicron spike was transiently expressed in HEK293T cells and purified with Strep-Tactin XT resin (IBA lifesciences). Plasmids encoding BA.1 RBD (319–541), BA.1 RBD (330–532) and BA.2 RBD (330–532) were transiently expressed in Expi293F™ Cells (ThermoFisher), cultured in FreeStyle™ 293 Expression Medium (ThermoFisher) at 30°C with 8% CO2 for 4 days. BA.1 RBD (330–532) was expressed in the presence of 1 μg/mL kifunensine. The harvested medium was concentrated using a QuixStand benchtop system. His-tagged ACE2 and RBDs were purified with a 5 mL HisTrap nickel column (GE Healthcare), followed by a Superdex 75 10/300 GL gel filtration column (GE Healthcare).

#### IgG mAbs and fab purification

Heavy and light chains of the indicated antibodies were transiently transfected into 293T cells. To purify full length IgG mAbs, supernatants of mAb expression were collected and filtered by a vacuum filter system and loaded on protein A/G beads overnight at 4°C. Beads were washed with PBS three times and 0.1 M glycine pH 2.7 was used to elute IgG. The eluate was neutralized with Tris-HCl pH 8 buffer to make the final pH = 7. The IgG concentration was determined by spectrophotometry and buffered exchanged into PBS.

Small amounts of Fab fragments were digested from purified IgGs with papain using a Pierce Fab Preparation Kit (Thermo Fisher), following the manufacturer’s protocol. AstraZeneca and Regeneron antibodies were provided by AstraZeneca, Vir, Lilly and Adagio antibodies were provided by Adagio.

To express and purify large amount of Fabs, heavy chain and light chain expression plasmids of each Fab were co-transfected into HEK293T cells by PEI. Cells were cultured for 5 days at 37 °C with 5% CO2, culture supernatant was harvested and filtered using a 0.22 mm polyethersulfone filter. Twin-strep tagged Fabs were purified using Strep-Tactin XT resin (IBA lifesciences). IgG Omi-18, Omi-31 and Omi-42 were transiently expressed in Expi293F™ Cells (ThermoFisher), cultured in FreeStyle™ 293 Expression Medium (ThermoFisher) at 30°C with 8% CO2 for 5 days. Purification was performed in the same way as other IgGs.

#### Nanobody production

The gene for nanobody C1 (NbC1) and F2 (NbF2) and were codon-optimized using the IDT Codon Optimization Tool, synthesized as a ready-to-clone gene fragment (Integrated DNA Technologies), and cloned into the phagemid vector pADL-23c. The nanobodies were produced as previously described (Huo et al., 2021). Briefly, the plasmid was transformed into the WK6 *E. coli* strain and protein expression induced by 1 mM IPTG grown overnight at 28°C. Periplasmic extract was prepared by osmotic shock, and the nanobody protein was purified with a 5 mL HisTrap nickel column (Cytiva), followed by size exclusion with a Hiload 16/60 Superdex 75 column.

#### Surface plasmon resonance

The surface plasmon resonance experiments were performed using a Biacore T200 (GE Healthcare). All assays were performed with a running buffer of HBS-EP (Cytiva) at 25°C. To determine the binding kinetics between the SARS-CoV-2 RBDs and ACE2 / monoclonal antibody (mAb), a Protein A sensor chip (Cytiva) was used. ACE2-Fc or mAb was immobilized onto the sample flow cell of the sensor chip. The reference flow cell was left blank. RBD was injected over the two flow cells at a range of five concentrations prepared by serial twofold dilutions, at a flow rate of 30 μL min^−1^ using a single-cycle kinetics program. Running buffer was also injected using the same program for background subtraction. All data were fitted to a 1:1 binding model using Biacore T200 Evaluation Software 3.1. To determine the binding kinetics between the SARS-CoV-2 Spikes and ACE2, a Twin-Strep-tag® Capture Kit (IBA-Lifesciences) was used. Spike protein containing a twin-Strep-tag was immobilized onto the sample flow cell of the sensor chip. The reference flow cell was left blank. ACE2 was injected over the two flow cells at a range of five concentrations prepared by serial twofold dilutions, at a flow rate of 30 μL min^−1^ using a single-cycle kinetics program. Running buffer was also injected using the same program for background subtraction. All data were fitted to a 1:1 binding model using Biacore T200 Evaluation Software 3.1.

#### Competition assays of anti-Omicron BA.1 RBD mAbs

Competition assays of anti-Omicron BA.1 RBD mAbs were performed on an Octet Red 96e machine (Sartorius) using Octet Anti-HIS (HIS2) Biosensors (Sartorius). His-tagged Omicron BA.2 RBD dissolved in the running buffer (10 mM HEPES, pH 7.4 and 150 mM NaCl) was used as the ligand and was first immobilized onto the biosensors. The biosensors were then washed with the running buffer to remove unbound RBD. Each biosensor was dipped into different saturating mAbs (Ab1) to saturate the bound RBD, except one biosensor was dipped into running buffer in this step, acting as the reference. Then all biosensors were washed with the running buffer again and dipped into wells containing the same competing antibody (Ab2). The y axis values of signals of different saturating antibodies in this step were divided by the value of the reference channel to get ratio results of different Ab1-Ab2 pairs. Ratio results close to 0 indicated total competition while 1 indicated no competition.

#### Crystallization

RBD proteins were deglycosylated with Endoglycosidase F1 before used for crystallization. Initial screening of crystals was set up in Crystalquick 96-well X plates (Greiner Bio-One) with a Cartesian Robot using the nanoliter sitting-drop vapor-diffusion method, with 100 nL of protein plus 100 nL of reservoir in each drop, as previously described (Walter et al., 2003).

For crystallization, Omicron BA.1-RBD was mixed with Omi-25 Fab, and Omicron BA.2-RBD was mixed with COVOX-150 and ACE2 separately, in a 1:1 molar ratio, with a final concentration of 13 mg mL^-1^. Omicron BA.1-RBD was mixed with Omi-3 and EY6A Fabs, Omi-6 and COVOX-150 Fabs, Omi-9 Fab and Nanobody F2 (NbF2), and Omi-12 and beta-54 Fabs separately, in a 1:1:1 molar ratio, with a final concentration of 7 mg mL^-1^. Omicron BA.1-RBD was mixed with Omi-32 Fab and NbC1 in a 1:1:1 molar ratio, with a final concentration of 11 mg/mL. Omi18 Fab, Omi31 Fab and NbC1 were mixed with Omicron BA.1-RBD and beta-RBD separately, in a 1:1:1:1 molar ratio, with a final concentration of 7 mg mL^-1^.These complexes were separately incubated at room temperature for 30 min. Omi-42 Fab was also crystallized.

Crystals of BA.1-RBD/Omi-25 were obtained from Molecular Dimensions Proplex condition 1–31, containing 3.0 M Sodium formate and 0.1 M Tris pH 7.5. BA.2-RBD/COVOX-150 crystals were obtained in 2 different space groups. Crystals of space group C2 were formed in Hampton Research PEGRx condition 1–29, containing 0.1 M Sodium citrate tribasic dihydrate pH 5.5 and 18% (w/v) PEG 3350. Crystals of space group P2_1_ were obtained from Hampton Research PEGRx condition 1–19, containing 0.1 M Sodium acetate trihydrate pH 4.5 and 30% (w/v) PEG 1500. Crystals of BA.2-RBD/ACE2 were formed in Hampton Research PEGRx condition 1–23, containing 0.1 M MES monohydrate pH 6.0 and 20% (w/v) PEG monomethyl ether 2000 and further optimized in 0.09 M MES monohydrate pH 6.0 and 18% (w/v) PEG monomethyl ether 2000. Crystals of BA.1-RBD/Omi-3/EY6A were formed in Hampton Research PEGRx condition 1–25, containing 0.1 M sodium citrate tribasic dihydrate pH 5.0 and 30% (v/v) Jeffamine® ED-2001 pH 7.0. Crystals of BA.1-RBD/Omi-6/COVOX-150 were obtained from Molecular Dimensions Proplex 1–23, containing 0.1 M Sodium HEPES pH 7.0 and 15% (w/v) PEG 4000. Crystals of BA.1-RBD/Omi-9/NbF2 were obtained from Hampton Research PEGRx condition 1–19, containing 0.1 M Sodium acetate trihydrate pH 4.5 and 30% (w/v) PEG 1500. Crystals of BA.1-RBD/Omi-12/beta-54 were formed in Hampton Research PEGRx condition 1–46, containing 0.1 M Sodium citrate tribasic dihydrate pH 5.0 and 18% (w/v) PEG 20000. Complex of BA.1-RBD/Omi-12/beta-54 was screen in Hampton Research Ammonium sulfate screen C2, containing 2.4 M (NH4)_2_SO4 and 0.1 M citric acid pH 5.0, but only crystals of Fab Omi-12 alone were formed in this condition. Crystals of BA.1-RBD/Omi-32/NbC1 were formed in Hampton Research PEGRx condition 2–35, containing 0.15 M Lithium sulfate monohydrate, 0.1 M Citric acid pH 3.5 and 18% (w/v) PEG 6000. Crystals of BA.1-RBD/Omi18/Omi31/NbC1 were formed in Molecular Dimensions Proplex condition 2–12, containing 0.2 M Ammonium sulfate, 0.1 M MES pH 6.5 and 20 % (w/v) PEG 8000. Crystals of beta-RBD/Omi18/Omi31/NbC1 were formed in Molecular Dimensions JCSG plus condition 1–48, containing 0.04 M Potassium phosphate monobasic and 16% (w/v) PEG 8000. Crystals of Omi-42 Fab alone were formed in Hampton Research PEGRx condition 1–24, containing 0.1 M Tris pH 8.0 and 30% (w/v) PEG monomethyl ether 2000.

#### X-ray data collection, structure determination and refinement

Diffraction data were collected at 100 K at beamline I03 of Diamond Light Source, UK, apart from data of BA.1 RBD/Omi-18-Omi-31-C1 and Beta RBD/Omi-18-Omi-31-C1 complexes, which were collected at beamline I04. All data were collected as part of an automated queue system allowing unattended automated data collection (https://www.diamond.ac.uk/Instruments/Mx/I03/I03-Manual/Unattended-Data-Collections.html). Crystals were pre-frozen by mounting in loops and soaked for a second in cryo-protectant containing 25% glycerol and 75% mother liquor. Diffraction images of 0.1° rotation were recorded on an Eiger2 XE 16M detector (exposure time from 0.015 to 0.026 s per image, beam size 80×20 μm, 10% beam transmission and wavelength of 0.9762 Å at I03; exposure time 0.22 s per image, beam size 63×50 μm, 100% beam transmission and wavelength of 0.9795 Å at I04). Data were indexed, integrated and scaled with the automated data processing program Xia2-dials (Winter, 2010; Winter et al., 2018). 720° of data was collected from 2 positions of a single crystal for BA.1 RBD/Omi-18-Omi-31-C1 complex, and 720° of data was collected for the P2_1_ crystal form of the Omicron BA.2-RBD/COVOX-150 complex from two crystals. 360° of data was collected from a single crystal for each of the other data sets.

Structures were determined by molecular replacement with PHASER(McCoy et al., 2007). VhVl and ChCl domains which have the most sequence similarity to previously determined SARS-CoV-2 RBD/Fab structures (Dejnirattisai et al., 2021a, 2021b; Huo et al., 2020; Liu et al., 2021a; Supasa et al., 2021; Zhou et al., 2020, 2021) were used as search models for each of the current structure determination. Model rebuilding with COOT (Emsley et al., 2010) and refinement with Phenix (Liebschner et al., 2019) were used for all the structures. Due to the lower resolution, only rigid-body and group B-factor refinement were performed for structures of Omicron BA.1-RBD/Omi-6-150, BA.1-RBD/Omi-9-NbF2, BA.1-RBD/Omi-12-Beta-54 and BA.2-RBD/ACE2 complexes. Crystals of Omicron RBD complexes tend to diffract weakly and to lower resolution. The N- and C-terminus of the RBD are flexible and have poor density. The ChCl domains in several complexes are also flexible with poorly defined density.

Data collection and structure refinement statistics are given in Table S3. Structural comparisons used SHP (Stuart et al., 1979), residues forming the RBD/Fab interface were identified with PISA (Krissinel and Henrick, 2007) and figures were prepared with PyMOL (The PyMOL Molecular Graphics System, Version 1.2r3pre, Schrödinger, LLC).

#### Cryo-EM grid preparation

A 3 μL aliquot of B.1.135 S ectodomain at a concentration of ∼1.2 μm with fab (1:6 molar ratio) was prepared, aspirated and almost immediately applied to a freshly glow-discharged C-flat 200 mesh 2/1 grids at high intensity, 20 s, Plasma Cleaner PDC-002-CE, Harrick Plasma. Excess liquid was removed by blotting for 5 s with a force of −1 using vitrobot filter paper (grade 595, Ted Pella Inc.) at 4.5°C, 100 % reported humidity before plunge freezing into liquid ethane using a Vitrobot Mark IV (Thermo Fisher). Fab/Spike complexes were incubated for 5–10 minutes prior to application to grids and plunge freezing.

#### Cryo-EM data collection

##### B.1.135 S ectodomain with Omi-2 fab

Movies were collected in mrc format using EPU on a 200 kV Glacios microscope equipped with a Falcon-III detector in linear mode, a 50 μm aperture, and 100 μm objective were employed. A total of 3269 movies were recorded with a total dose of 45 e/Å2 and a pixel size 1.2 Å/pix with fringe free illumination.

##### B.1.135 S ectodomain with Omi-38 or Omi-42 fab

Compressed tiff movies, 8084 and 5638 respectively, each with 40 frames, were acquired on a Titan Krios (Thermo Fisher) operating at 300 kV with a K3 detector and 20 eV slit (Gatan) at a nominal magnification of 105 kX in super resolution mode (corresponding to a calibrated pixel size of 0.415 Å/pix at super resolution). A total dose of 50.5 e/Å^2^ was applied to each movie and defocus range of 0.8–2.6 μm.

#### Cryo-EM data processing

For all three datasets, movies were 4-times binned and motion and ctf corrected on the fly using the cryoSPARC v3.3.1 live framework (Punjani et al., 2017). Particles were initially picked with the blob-picker module before spike-like particles from 2D classification of this initial set were used as a template for template-based picking. Maps and FSC curves for all analyses are shown in Figure S4I. For Omi-42 particles were sorted in two rounds of 2D classification followed by ab-initio reference classification into three classes, followed by a second classification into two classes. Particles from the best class, 106811 in total, were then further refined to 3.64 Å reported resolution (as determined within the cryoSPARC interface, AuFSC = 0.143). A second, somewhat lower resolution class, where RBDs were oriented slightly differently was also refined (see Figure S4I). For Omi-2 182828 particles were derived from two rounds of classification, before further 3D classification and local refinement of the entire spike, but with the fulcrum focused at the RBD/fab region to better resolve the interfaces of interest (various local refinements with masking and with/without subtracted densities failed to improve this region). For Omi-38, particles were sorted in two rounds of 2D classification before classification using three ab-initio models. The best class, with 201474 particles was then refined further, with global and local ctf refinement and no symmetry imposed, resulting in a final reported global reconstruction at AuFSC 0.143 of 2.90 Å (as determined within the cryoSPARC interface (Punjani et al., 2017)). Local refinement of Omi-38 with B.1.135 was performed also using cryoSPARC upon this particle set from which the areas outside of the area of interest (two upwards conformation RBDs in closeness, proximity to each other and associated fabs) was subtracted. Areas were subtracted/refined using masks created in Chimera X (Pettersen et al., 2021). Masks were created as follows, within Chimera X, the area of interest was selected from the global spike map using the volume eraser tool, a Gaussian filter was then applied, and the resulting volume imported into cryoSPARC with an additional dilation radius of 5 and soft padding width of 5 pixels. The final reconstruction from local refinement was reportedly at a resolution of AuFSC 0.143 3.69 Å (as determined within the cryoSPARC interface) and clearly enhanced the variable domain/RBD interface.

#### Antibody mapping to RBD surface

All Omicron antibodies and antibodies with previously solved structures (COVOX-45, -58, −222, EY6A and beta-54) were used in a competition assay prepared for antibody mapping to the RBD surface. Antibody mapping was carried out using *mabscape* (Dejnirattisai et al., 2021a) and cluster4x (Ginn, 2020). Mid-point positions of EY6A, COVOX-45, COVOX-222 and beta-54 were calculated from crystal structures and used to seed the analysis in 1000 Monte Carlo runs, whereas known structural positions of Omi-3, Omi-9, Omi-12 and COVOX-58 were not included in the analysis and used as a cross-check. A total of 178 Monte Carlo runs formed a single cluster with the lowest score and these were used to calculate average positions for Omicron antibodies.

### Quantification and statistical analysis

Statistical analyses are reported in the results and figure legends. Neutralization was measured by FRNT. The percentage of focus reduction was calculated and IC_50_ (FRNT50) was determined using the probit program from the SPSS package. The Wilcoxon matched-pairs signed rank test was used for the analysis and two-tailed P values were calculated on geometric mean values.

## Data Availability

The coordinates and structure factors of the crystallographic complexes are available from the PDB with accession codes listed in Table S3. Mabscape is available from https://github.com/helenginn/mabscape, https://snapcraft.io/mabscape. The data that support the findings of this study are available from the corresponding authors on request.

## Supplemental information

Document S1. Tables S1, S2A, S2B, S3A, and S3B
