# Supplementary material for: Potent cross-reactive antibodies following Omicron breakthrough in vaccinees
Source: Cell. 2022 Jun 9;185(12):2116–2131.e18. doi: 10.1016/j.cell.2022.05.014 (PMC9120130; doi:10.1016/j.cell.2022.05.014)
Supplement: Document S1. Tables S1, S2A, S2B, S3A, and S3B [file mmc1.pdf]

Cell, Volume *185*

Supplemental information

Potent cross-reactive antibodies following Omicron breakthrough in vaccinees

Rungtiwa Nutalai, Daming Zhou, Aekkachai Tuekprakhon, Helen M. Ginn, Piyada Supasa, Chang Liu, Jiandong Huo, Alexander J. Mentzer, Helen M.E. Duyvesteyn, Aiste Dijokaite-Guraliuc, Donal Skelly, Thomas G. Ritter, Ali Amini, Sagida Bibi, Sandra Adele, Sile Ann Johnson, Bede Constantinides, Hermione Webster, Nigel Temperton, Paul Klenerman, Eleanor Barnes, Susanna J. Dunachie, Derrick Crook, Andrew J. Pollard, Teresa Lambe, Philip Goulder, OPTIC consortium, ISARIC4C consortium, Neil G. Paterson, Mark A. Williams, David R. Hall, Juthathip Mongkolsapaya, Elizabeth E. Fry, Wanwisa Dejnirattisai, Jingshan Ren, David I. Stuart, and Gavin R. Screaton

Table S1. Omicron mAbs heavy and light chain variable gene usage and mutation analysis. Related to Figure 2.

mAbs	Patient No.	Heavy chain				Light chain			
		V-GENE	J-GENE	D-GENE	#Amino acid substitutions	K/λ	V-GENE	J-GENE	#Amino acid substitutions
Omi-02	07	1-69*01 , or 1-69D*01	2*01	2-21*02	7	K	3-20*01	5*01	5
Omi-03	07	3-53*01	4*02	1-26*01	5	K	3-20*01	2*01	0
Omi-06	07	4-4*07	3*02	3-16*02	4	K	1-39*01 , or 1D-39*01	4*01	12
Omi-08	07	1-46*01 , or 1-46*03	4*02	6-13*01	12	λ	1-40*02	1*01	4
Omi-09	07	3-30*01	3*02	4-17*01	6	λ	3-25*02	2*01 , or 3*01	4
Omi-12	08	1-58*02	3*02	2-2*01	12	K	3-20*01	1*01	9
Omi-16	09	3-66*02	4*02	2-15*01	9	K	3-20*01	2*01	7
Omi-17	09	3-66*02	4*02	6-19*01	7	K	3-20*01	2*01	6
Omi-18	09	3-53*01	6*02	4-11*01	11	λ	3-21*02	1*01	6
Omi-20	09	3-66*02	6*02	5-12*01	11	K	1-9*01	4*02	5
Omi-23	12	4-31*03	4*02	3-22*01	6	K	1-NL1*01	1*01	5
Omi-24	14	1-69*06	4*02	3-16*02	9	K	3-15*01	1*01	7
Omi-25	14	3-9*01	6*02	3-16*01	6	K	1-39*01 , or 1D-39*01	2*01	7
Omi-26	14	1-18*01	4*02	1-26*01	12	λ	1-36*01	3*02	4
Omi-27	14	3-66*01 , or 3-66*04	6*02	6-19*01	8	K	1-6*01	2*01	6
Omi-28	14	3-66*01 , or 3-66*04	4*02	3-16*01	4	K	3-20*01	1*01	9
Omi-29	14	3-53*04	6*02	2-15*01	11	λ	2-14*01 , or 2-14*03	3*02	6
Omi-30	14	1-69*06	6*02	2-15*01	10	λ	1-44*01	3*02	7
Omi-31	14	1-69*06	6*02	3-16*01	11	λ	1-44*01	3*02	6
Omi-32	08	3-33*01 , or 3-33*06	4*02	2-21*02	6	K	3-20*01	4*01	6
Omi-33	08	3-33*01 , or 3-33*06	4*02	2-21*02	10	K	3-20*01	4*01	4
Omi-34	09	1-69*06 , or 1-69*14	4*02	2-2*01	10	λ	1-40*01	1*01	6
Omi-35	09	3-9*01	6*02	2-2*02	5	λ	3-21*02	2*01 , or 3*01	7
Omi-36	09	3-66*02	4*02	2-15*01	9	K	3-20*01	2*01	5
Omi-38	15	1-69*09	3*01	1-26*01	16	K	1-5*01	5*01	10
Omi-39	07	3-43*01	6*03	2-2*01	8	K	4-1*01	3*01	6
Omi-41	08	1-18*04	4*02	3-9*01	11	K	4-1*01	2*02 ()	5
Omi-42	09	3-9*01	6*02	6-19*01	7	λ	2-8*01	2*01 , or 3*01 or 3*02	5

Table S2A. VoC and Omicron neutralization data for Omicron mAbs. Related to Figure 3.

mAbs	IC50 (ug/ml)							
	Victoria	Alpha	Beta	Gamma	Delta	BA.1	BA.1.1	BA.2
**Omi-02**	0.015 ± 0.001	0.014 ± 0.005	0.009 ± 0.000	0.004 ± 0.000	0.014 ± 0.003	0.013 ± 0.001	0.015 ± 0.001	0.040 ± 0.021
**Omi-03**	0.007 ± 0.000	0.012 ± 0.007	0.009 ± 0.001	0.004 ± 0.000	0.004 ± 0.000	0.009 ± 0.002	0.015 ± 0.000	0.028 ± 0.002
**Omi-06**	0.007 ± 0.001	0.011 ± 0.002	0.012 ± 0.000	0.010 ± 0.003	5.040 ± 0.747	0.054 ± 0.005	1.505 ± 0.341	0.238 ± 0.007
**Omi-08**	0.014 ± 0.007	0.022 ± 0.002	0.007 ± 0.000	0.024 ± 0.007	0.048 ± 0.012	0.008 ± 0.004	0.007 ± 0.001	1.510 ± 0.683
**Omi-09**	0.004 ± 0.001	0.002 ± 0.000	1.218 ± 0.324	2.373 ± 1.008	0.008 ± 0.002	0.011 ± 0.005	0.017 ± 0.003	0.034 ± 0.010
**Omi-12**	0.005 ± 0.000	0.003 ± 0.001	0.006 ± 0.001	0.003 ± 0.000	0.003 ± 0.000	0.004 ± 0.001	0.009 ± 0.001	0.010 ± 0.001
**Omi-16**	0.016 ± 0.002	0.022 ±0.009	0.018 ±0.004	0.022 ±0.007	0.016 ±0.002	0.019 ±0.003	0.027 ± 0.007	0.067 ± 0.021
**Omi-17**	0.066 ± 0.015	0.098 ±0.027	0.021 ±0.007	0.021 ±0.007	0.074 ±0.019	0.028 ±0.005	0.026 ± 0.001	0.095 ± 0.008
**Omi-18**	0.041 ± 0.005	0.038 ±0.008	0.018 ±0.006	0.016 ±0.004	0.025 ±0.000	0.006 ±0.003	0.006 ± 0.001	0.007 ± 0.001
**Omi-20**	0.012 ± 0.002	0.023 ±0.004	0.019 ±0.009	0.019 ±0.006	0.008 ±0.001	0.043 ±0.012	0.032 ± 0.002	0.022 ± 0.005
**Omi-23**	0.005 ± 0.002	0.009 ±0.004	0.020 ±0.005	0.018 ±0.006	0.006 ±0.002	0.044 ±0.013	0.03 ± 0.001	0.028 ± 0.001
**Omi-24**	0.005 ± 0.001	0.008 ±0.003	0.006 ±0.001	0.010 ±0.005	>10	0.007 ±0.001	0.035 ± 0.010	0.008 ± 0002
**Omi-25**	0.003 ± 0.001	0.007 ±0.001	0.059 ±0.007	0.257 ±0.079	0.006 ±0.002	0.046 ±0.015	0.138 ± 0.046	0.056 ± 0.030
**Omi-26**	0.005 ± 0.000	0.010 ±0.003	0.055 ±0.020	0.214 ±0.046	0.005 ±0.001	0.034 ±0.000	0.055 ± 0.030	0.03 ± 0.011
**Omi-27**	0.026 ± 0.001	0.032 ±0.012	0.019 ±0.006	0.017 ±0.006	0.010 ±0.001	0.091 ±0.050	0.239 ± 0.052	0.039 ± 0.006
**Omi-28**	0.028 ± 0.004	0.028 ±0.001	0.019 ±0.010	0.033 ±0.008	0.018 ±0.002	0.032 ±0.009	0.075 ± 0.032	0.047 ± 0.010
**Omi-29**	0.044 ± 0.002	0.066 ±0.034	0.048 ±0.020	0.040 ±0.007	0.029 ±0.004	0.036 ±0.003	0.052 ± 0.004	0.192 ± 0.021
**Omi-30**	0.109 ± 0.035	0.043 ±0.016	0.028 ±0.009	0.038 ±0.004	>10	0.058 ±0.008	0.084 ± 0.021	0.045 ± 0.010
**Omi-31**	0.007 ± 0.001	0.020 ±0.003	0.011 ±0.005	0.017 ±0.006	>10	0.010 ±0.002	0.017 ± 0.009	0.083 ± 0.040
**Omi-32**	0.032 ± 0.016	0.102 ±0.041	0.460 ±0.092	0.430 ±0.012	0.012 ±0.002	0.024 ±0.011	4.642 ± 0.283	1.899 ± 0.280
**Omi-33**	0.028 ± 0.005	0.057 ± 0.017	0.136 ± 0.002	0.132 ± 0.037	0.011 ± 0.001	0.026 ± 0.008	0.113 ± 0.035	0.681 ± 0.0170
**Omi-34**	0.003 ± 0.001	0.041 ±0.027	0.003 ±0.000	0.008 ±0.002	>10	0.028 ±0.009	0.074 ± 0.016	0.014 ± 0.003
**Omi-35**	0.057 ± 0.003	0.080 ±0.030	0.128 ±0.058	0.136 ±0.024	0.280 ±0.059	0.069 ±0.032	0.262 ± 0.086	0.082 ± 0.043
**Omi-36**	0.056 ± 0.008	0.047 ± 0.009	0.018 ± 0.001	0.015 ± 0.000	0.026 ± 0.003	0.038 ± 0.006	0.053 ± 0.022	0.105 ± 0.023
**Omi-38**	0.001 ± 0.000	0.009 ± 0.001	0.004 ± 0.000	0.002 ± 0.000	0.004 ± 0.001	0.054 ± 0.028	>10	0.027 ± 0.001
**Omi-39**	0.015 ± 0.006	0.039 ± 0.007	0.009 ± 0.000	0.014 ± 0.001	0.012 ± 0.007	0.025 ± 0.004	>10	0.073 ± 0.014
**Omi-41**	0.090 ± 0.013	2.262 ± 1.199	>10	0.126 ± 0.059	>10	0.081 ± 0.004	0.191 ± 0.014	>10
**Omi-42**	0.016 ± 0.003	0.024 ± 0.001	0.011 ± 0.004	0.013 ± 0.003	0.019 ± 0.001	0.014 ± 0.002	0.017 ± 0.004	0.031 ± 0.008
**REGN10987**	0.032 ± 0.007	0.028 ± 0.003	0.007 ± 0.001	0.013 ± 0.002	0.017 ± 0.009	>10	>10	1.847 ± 1.231
**REGN10933**	0.004 ± 0.002	0.014 ± 0.002	3.284 ± 2.014	6.177 ± 1.914	0.003 ± 0.001	>10	>10	>10
**AZD1061**	0.013 ± 0.003	0.012 ± 0.002	0.014 ± 0.002	0.007 ± 0.002	0.038 ± 0.006	3.488 ± 2.085	>10	0.028 ± 0.014
**AZD8895**	0.005 ± 0.001	0.011 ± 0.002	0.046 ± 0.031	0.046 ± 0.016	0.003 ± 0.000	1.152 ± 0.170	6.078 ± 1.558	7.702 ± 2.224
**AZD7442**	0.009 ± 0.000	0.007 ± 0.001	0.012 ± 0.001	0.006 ± 0.003	0.005 ± 0.000	0.273 ± 0.062	3.816 ± 0.138	0.052 ± 0.004
**ADG10**	0.006 ± 0.000	0.010 ± 0.001	0.011 ± 0.001	0.003 ± 0.000	0.026 ± 0.005	>10	>10	>10
**ADG20**	0.004 ± 0.001	0.006 ± 0.000	0.01 ± 0.001	0.009 ± 0.000	0.006 ± 0.001	1.104 ± 0.509	1.269 ± 0.223	>10
**ADG30**	0.007 ± 0.002	0.016 ± 0.001	0.029 ± 0.003	0.002 ± 0.001	0.033 ± 0.007	>10	>10	>10
**Ly-CoV-555**	0.006 ± 0.002	0.009 ± 0.000	>10	>10	8.311 ± 4.059	>10	>10	>10
**Ly-CoV16**	0.034 ± 0.007	3.225 ± 1.030	>10	>10	0.012 ± 0.002	>10	>10	>10
**S309**	0.040 ± 0.005	0.078 ± 0.069	0.082 ± 0.002	0.076 ± 0.014	0.113 ± 0.028	0.256 ± 0.034	1.119 ± 0.119	5.035 ± 0.244

Table S2B. Neutralization data for early pandemic and Beta mAbs. Related to Figure 3.

Early pandemic mAbs	IC50 (ug/ml)			
	Victoria	BA.1	BA.1.1	BA.2
40	0.006 ± 0.002	1.705 ± 0.840	0.544 ± 0.007	0.100 ± 0.007
55	0.006 ± 0.002	>10	>10	>10
58	0.019 ± 0.004	0.060 ± 0.041	0.876 ± 0.135	0.043 ± 0.007
88	0.005 ± 0.002	>10	>10	>10
132	0.012 ± 0.004	>10	>10	>10
150	0.008 ± 0.004	>10	3.500 ± 0.712	>10
158	0.021 ± 0.006	>10	2.843 ± 0.733	4.249 ± 0.694
159	>10	>10	>10	>10
165	0.007 ± 0.005	>10	>10	>10
170	0.006 ± 0.001	>10	>10	>10
175	0.012 ± 0.004	>10	>10	>10
222	0.006 ± 0.000	0.021 ± 0.002	0.023 ± 0.001	0.249 ± 0.082
253	0.021 ± 0.009	0.875 ± 0.373	0.415 ± 0.161	1.100 ± 0.049
269	0.008 ± 0.004	>10	>10	>10
278	0.001 ± 0.000	>10	>10	0.326 ± 0.011
281	0.001 ± 0.000	>10	>10	>10
316	0.001 ± 0.000	>10	>10	>10
318	0.012 ± 0.003	9.490 ± 4.540	>10	0.303 ± 0.190
384	0.001 ± 0.000	>10	>10	>10
398	0.072 ± 0.065	>10	>10	>10
253+55	0.001 ± 0.000	0.638 ± 0.315	0.451 ± 0.014	>10
253+165	0.001 ± 0.000	>10	6.591 ± 0.799	>10

Beta mAbs	IC50 (ug/ml)			
	Beta	BA.1	BA.1.1	BA.2
β06	0.005 ± 0.001	>10	>10	>10
β10	0.021 ± 0.008	>10	>10	>10
β20	0.006 ± 0.002	5.679 ± 0.452	1.836 ± 0.780	>10
β22	0.041 ± 0.014	0.479 ± 0.029	0.130 ± 0.005	>10
β23	0.005 ± 0.001	>10	>10	>10
β24	0.002 ± 0.000	>10	>10	>10
β26	0.004 ± 0.001	>10	>10	>10
β27	0.003 ± 0.001	0.766 ± 0.043	0.274 ± 0.095	0.348 ± 0.030
β29	0.009 ± 0.000	0.095 ± 0.029	0.066 ± 0.002	4.029 ± 0.402
β30	0.002 ± 0.000	>10	>10	>10
β32	0.023 ± 0.001	>10	>10	>10
β33	0.020 ± 0.002	>10	>10	>10
β34	0.030 ± 0.004	>10	>10	>10
β38	0.004 ± 0.001	>10	>10	>10
β40	0.001 ± 0.000	0.005 ± 0.001	0.002 ± 0.000	0.008 ± 0.002
β43	0.014 ± 0.003	>10	>10	>10
β44	0.008 ± 0.001	>10	>10	>10
β45	0.010 ± 0.001	>10	>10	>10
β47	0.002 ± 0.000	0.018 ± 0.009	0.011 ± 0.002	0.044 ± 0.006
β48	0.003 ± 0.001	5.706 ± 0.676	0.752 ± 0.052	5.042 ± 0.650
β49	0.014 ± 0.004	>10	>10	>10
β50	0.008 ± 0.001	>10	>10	>10
β51	0.003 ± 0.000	>10	>10	>10
β53	0.007 ± 0.001	0.141 ± 0.026	5.849 ± 0.036	0.170 ± 0.073
β54	0.002 ± 0.000	0.003 ± 0.001	0.001 ± 0.000	0.076 ± 0.029
β55	0.009 ± 0.002	0.033 ± 0.008	0.009 ± 0.001	0.069 ± 0.008

Table S3A. X-ray data collection and structure refinement statistics. Related to Figures 5-7.

**Structure**	BA.1 RBD/ Omi3-EY6A	BA.1 RBD/ Omi9-NbF2	BA.1 RBD/ Omi12-Beta54[a]	Omi12 Fab[a]	BA.2 RBD/ ACE2	BA.2 RBD/150	BA.2 RBD/150	BA.1 RBD/ Omi6-150	BA.1 RBD/ Omi18-Omi31-NbC1	Beta RBD/ Omi18-Omi31-NbC1	BA.1 RBD/ Omi25	BA.1 RBD/ Omi-32-NbC1	Omi42 Fab
PDB ID	7ZF3	7ZF4	7ZF5	7ZF6	7ZF7	7ZF8	7ZF9	7ZFA	7ZFB	7ZFC	7ZFD	7ZFE	7ZFF
**Data collection**													
Space group	*P*212121	*C*2221	*P*21	*C*2221	*P*41212	*C*2	*P*21	*P*1	*P*21	*P*3121	*P*43212	P21	*P*21
Cell dimensions													
a, *b*, *c* (Å)	87.5, 119.9, 134.0	86.6, 205.1, 123.1	95.7, 156.3, 122.4	65.0, 210.1, 85.9	104.2, 104.2, 223.7	194.2, 94.9, 58.4	90.0, 83.9, 110.7	82.8, 114.8, 144.6	111.2, 135.1, 112.2	105.0, 105.0, 234.5	123.0, 123.0, 223.7	98.5, 159.9, 133.3	63.8, 49.4, 72.3
α,β,γ (°)	90, 90, 90	90, 90, 90	90, 90.3, 90	90, 90, 90	90, 90, 90	90, 101.0, 90	90, 102.0, 90	82.0, 80.6, 86.2	90, 101.7, 90	90, 90, 120	90, 90, 90	90, 106.9, 90	90, 115.6, 90
Resolution (Å)	73–3.15 (3.20-3.15)[b]	77–4.18 (4.25-4.18)	78–5.50 (5.60-5.50)	53–2.08 (2.12-2.08)	76–3.46 (3.52-3.46)	95–2.95 (3.00-2.95)	66–3.25 (3.30-3.25)	114–4.24 ((4.74-4.24)	87–3.08 (3.13-3.08)	85–3.24 (3.29-3.24)	69–3.39 (3.45-3.39)	81–3.25 (3.76-3.25)	65–2.32 (2.36-2.32)
*R*merge	0.491 (---)	0.728 (---)	0.641 (---)	0.179 (---)	0.703 (---)	0.490 (---)	0.629 (---)	0.330 (---)	0.264 (---)	0.265 (---)	--- (---)	0.241 (---)	0.182 (---)
*R*pim	0.136 (0.932)	0.207 (1.34)	0.259 (0.919)	0.052 (1.151)	0.138 (1.560)	0.198 (0.916)	0.173 (1.172)	0.205 (0.503)	0.073 (1.623)	0.073 (0.946)	0.233 (1.175)	0.097 (0.527)	0.078 (1.082)
I/ (*I*)	5.0 (0.6)	2.5 (0.4)	2.1 (0.4)	6.2 (0.2)	3.1 (0.3)	2.1 (0.3)	3.2 (0.4)	2.6 (1.6)	5.0 (0.2)	6.6 (0.3)	2.4 (0.4)	6.6 (1.6)	8.0 (0.5)
*CC*1/2	0.955 (0.328)	0.877 (0.340)	0.849 (0.332)	0.994 (0.255)	0.992 (0.317)	0.939 (0.303)	0.971 (0.428)	0.918 (0.590)	0.997 (0.286)	0.995 (0.298)	0.967 (0.314)	0.995 (0.474)	0.993 (0.348)
Completeness (%)	100 (99.2)	100 (98.3)	100 (98.2)	93.3 (62.9)	99.6 (99.3)	100 (99.8)	99.9 (97.9)	82.9 (74.8)	100 (100)	100 (98.4)	100 (99.7)	87.8 (49.0)	92.6 (54.7)
Redundancy	13.7 (14.2)	13.4 (13.1)	7.1 (7.4)	12.1 (6.8)	26.4 (27.8)	7.0 (6.7)	14.2 (14.7)	3.5 (3.5)	14.1 (13.5)	14.1 (14.3)	26.8 (28.3)	7.1 (7.0)	6.2 (3.7)
**Refinement**													
Resolution (Å)	73–3.15	62–4.18[c]	78–5.50[c]	53–2.08	76–3.46[c]	57–2.95	62–3.25	114–4.24[c]	71–3.08	85–3.24	69–3.39	81–3.25	65–2.32
No. reflections	23771/1232	7769/415	11051/615	29710/1547	15216/802	18383/976	24438/1205	11920/595	50808/2718	23355/1202	23272/1216	26894/1395	15619/865
*R*work / *R*free	0.208/0.266	0.369/0.385	0.284/0.283	0.235/0.265	0.258/0.269	0.220/0.258	0.199/0.231	0.237/0.273	0.261/0.308	0.244/0.299	0.286/0.338	0.237/0.293	0.231/0.269
Protein atoms #	8063	5757	16328	3320	6420	4798	9611	32080	17908	8914	9605	22831	3231
Ligand/ion/water #	32			133	85		28						109
Protein *B* factors (Å^2)	79	192	248	59	126	56	94	104	178	143	100	99	49
Ligand/ion/water *B* factors (Å^2)	74			74	149		135						51
RMSD Bond length (Å)	0.002	0.008	0.010	0.002	0.002	0.002	0.002	0.008	0.004	0.003	0.003	0.002	0.002
RMSD Bond angles (°)	0.5	1.0	0.7	0.6	0.5	0.5	0.5	1.2	0.7	0.5	0.6	0.6	0.5

[a] Omi12 is glycosylated at N102 of the heavy chain. [b] Values in parentheses are for highest-resolution shell. [c] Rigid body and group B-factor refinement only.

Table S3B. Cryo-EM data collection, refinement and validation statistics of spike/Fab complexes. Related to Figures 5-7.

	**Omi-2/Beta Spike ectodomain**	**Omi-38/Beta Spike ectodomain**	**Omi-38/Beta Spike RBD (local refinement)**	**Omi-42/Beta Spike ectodomain**
PDB ID / EMDB ID	7ZR9/EMD-14887	7ZRC, EMD-14910	7ZR8/EMD-14886	7ZR7/EMD-14885
**Data collection and processing**				
Microscope, Detector, Mode	Glacios, Falcon-III, linear	Krios, K3, superresolution	Krios, K3, superresolution	Krios, K3, superresolution
Voltage / kV	200	300	300	300
Electron exposure (e$^-$/Å^2)	50.0	50.2	50.2	50.2
Defocus Range (um)	-1.5 to -3.5 [0.5]	-0.8 to -2.6 [0.3]	-0.8 to -2.6 [0.3]	-0.8 to -2.6 [0.3]
Nominal magnification kX	92	105	105	105
Pixel size [super res] (Å)	1.22	0.83	0.83	0.83
Symmetry imposed	C1	C1	C1	C1
Particles in final reconstruction	182,828	201,474	201,474	106,884
Map resolution in Å (	4.0	2.9	3.7	3.6
FSC threshold	0.143	0.143	0.143	0.143
**Refinement**				
Initial model	7Q9G Spike, Alphafold Fab	7Q9G Spike Alphafold Fab	7Q9G Spike Alphafold Fab	7Q9G Spike Alphafold Fab
RBD conformation	Two-up, one poorly resolved, two clearly decorated.	Two mostly up, one tilted.	Two mostly up, one tilted, all decorated, but less clear for tilted.	Three-up, all decorated.
FSC threshold	0.143	0.143	0.143	0.143
Map sharpening B factor (Å^2)	-193	-69	-106	-95
Model non-hydrogen atoms	31014	30724	3693	30767
Protein residues	3899	3852	471	3878
Ligands	54	52	1	54
Protein mean *B* factor (Å^2)	238.1	30.3	31.2	213.7
Ligand mean *B* factor (Å^2)	130.9	42.9	21.8	167.6
RMSD Bond lengths (Å)	0.006	0.006	0.003	0.003
RMSD Bond angles (°)	0.857	0.596	0.458	0.496
Correlation Coefficient	0.76	0.75	0.82	0.79
**Validation**				
MolProbity score	1.76	1.61	1.65	1.58
Clashscore	8.11	7.36	7.02	6.01
Poor rotamers (%)	1.22	0.93	0.74	0.82
Ramachandran plot				
Favored (%)	96.2	96.7	96.1	96.3
Allowed (%)	3.69	3.3	3.9	3.7
Disallowed (%)	0.1	0.0	0.0	0.03
